# Quantification of variation and the impact of biomass in targeted 16S rRNA gene sequencing studies

**DOI:** 10.1186/s40168-018-0543-z

**Published:** 2018-09-10

**Authors:** Jeffrey M. Bender, Fan Li, Helty Adisetiyo, David Lee, Sara Zabih, Long Hung, Thomas A. Wilkinson, Pia S. Pannaraj, Rosemary C. She, Jennifer Dien Bard, Nicole H. Tobin, Grace M. Aldrovandi

**Affiliations:** 1 USC Keck School of Medicine and Children’s Hospital Los Angeles, 4650 Sunset Blvd, Los Angeles, CA 90027 USA; 2 Children’s Hospital Los Angeles, 4650 Sunset Blvd, Los Angeles, CA 90027 USA; 3 David Geffen School of Medicine at UCLA, 10833 Le Conte Ave, Los Angeles, CA 90095 USA; 4 USC Keck School of Medicine, 1975 Zonal Ave, Los Angeles, CA 90033 USA

**Keywords:** Biomass, Technical variation, Biological variation, Precision, Accuracy

## Abstract

**Background:**

Recent advances in sequencing technologies and bioinformatics tools have allowed for large-scale microbiome studies that are rapidly advancing medical research. However, small changes in technique or analysis can significantly alter the results and lead to conflicting findings. Quantifying the technical versus biological variation expected in targeted 16S rRNA gene sequencing studies and how this variation changes with input biomass is critical to guide meaningful interpretation of the current literature and plan future research.

**Results:**

Data were compiled from 469 sequencing libraries across 19 separate targeted 16S rRNA gene sequencing runs over a 2.5-year time period. Following removal of contaminant sequences identified from negative controls, 244 samples retained sufficient reads for further analysis. Coefficients of variation for intra- and inter-assay variation from repeated measurements of a bacterial mock community ranged from 8.7 to 37.6% (intra) and 15.6 to 80.5% (inter) for all but one genus of bacteria whose relative abundance was greater than 1%. Intra- versus inter-assay Bray-Curtis pairwise distances for a single stool sample were 0.11 versus 0.31, whereas intra-assay variation from repeat stool samples from the same donor was greater at 0.38 (Wilcoxon *p* = 0.001). A dilution series of the bacterial mock community was used to assess the effect of input biomass on variability. Pairwise distances increased with more dilute samples, and estimates of relative abundance became unreliable below approximately 100 copies of the 16S rRNA gene per microliter. Using this data, we created a prediction model to estimate the expected variation in microbiome measurements for given input biomass and relative abundance values.

**Conclusions:**

Well-controlled microbiome studies are sufficiently robust to capture small biological effects and can achieve levels of variability consistent with clinical assays. Relative abundance is negatively associated with measures of variability and has a stronger effect on variability than does absolute biomass, suggesting that it is feasible to detect differences in bacterial populations in very low-biomass samples. Further, by quantifying the effect of biomass and relative abundance on compositional variability, we developed a tool for defining the expected variance in a given microbiome study.

**Electronic supplementary material:**

The online version of this article (10.1186/s40168-018-0543-z) contains supplementary material, which is available to authorized users.

## Background

Continued improvements and decreasing costs of DNA sequencing technologies have enabled exponential growth in human microbiome studies [1]. Alterations in these complex and dynamic microbial communities have been associated with a plethora of human health-associated outcomes including obesity, autism, asthma, and inflammatory bowel disease. There is considerable enthusiasm for using targeted sequencing of microbial communities to understand disease pathogenesis as well as the development of novel diagnostics, therapeutics, and preventative measures. However, as with any relatively new technique, contradictory findings are present. For example, the presence of low-biomass bacterial communities in environments long thought to be sterile has been a topic of much debate, as these have been attributed to either reagent contamination or a true commensal population [2–5]. A growing body of work has demonstrated the importance of experimental design and execution on reproducibility and robustness of microbiome studies [6–9]. Differences in sample storage, DNA extraction method, primer choice, and laboratory conditions have all been shown to effect changes in the inferred microbial composition that lead to potentially conflicting associations with clinical outcomes [10–18].

Our goal in presenting this data is to help predict the reproducibility of the relative abundance of bacterial communities in microbiome studies using a standardized approach. In clinical assays, reproducibility relies on both precision and accuracy. Accuracy is difficult to measure in the absence of a well-defined ground truth, and most complex microbial communities remain as of yet too unevenly characterized to yield a true gold standard. As such, we focus here on measures of precision. Our group standardized all of the aforementioned experimental conditions and performed a large number of microbiome studies over the past few years. Through repeat sampling of both synthetic and biologic bacterial communities, we focus on quantifying the technical and biological variation that can be expected from rigorous microbiome experiments.

Specifically, through this study, we describe the baseline intra- and inter-assay variation from repeated measurements of a bacterial mock community across 19 sequencing runs over the course of approximately 2 years. We also examined intra- and inter-assay variation versus biological variation by repeated profiling of three stool specimens provided by a single donor. Finally, we quantified the robustness of amplicon-based microbiome profiling as a function of input bacterial biomass. We then defined a model combining relative abundance and concentration to help predict variance. These data show that the technical variation in well-controlled microbiome studies is similar to those of other standard laboratory assays. Additionally, these data provide novel insights into the effect of input biomass and relative abundance on variation in the inferred microbiome composition and may help guide future studies of similar low-input communities.

## Results

### Study design

Data were aggregated from 469 sequencing libraries across 19 different MiSeq runs (Table 1). All of the samples were subjected to the same DNA extraction, PCR amplification, and library preparation methods. To quantify intra- and inter-assay variation, data were analyzed from 118 libraries prepared from independent aliquots of a whole-cell bacterial mock community that was extracted and sequenced alongside other projects over the course of 2 years. To investigate biological versus technical variation, a set of 29 libraries was analyzed from three separate stool samples from a single donor at 2-week intervals. To assess the variance in sequencing low-biomass samples, given the stochastic variation inherent during PCR amplification of low quantities of DNA, a set of dilutions ranging from stock concentration to thousandfold dilutions of our mock community was performed and analyzed. Quantitative polymerase chain reaction (qPCR) was used to measure absolute 16S rRNA gene copy number followed by targeted 16S rRNA gene sequencing to determine microbial composition. With these data, a model was created to predict variation based on relative abundance and biomass.

**Table 1:** Overview of 19 sequencing runs with bacterial mock positive controls, repeat stool samples, and negative controls over a 2.5-year time period. All samples were processed in the same manner and run on the same sequencing machine. Run 18 includes a prospective dilution study of the mock bacterial controls. Numbers in parenthesis include the samples initially in analysis before negative control filtering

Sample type	Run 1	Run 2	Run 3	Run 4	Run 5	Run 6	Run 7	Run 8	Run 9	Run 10	Run 11	Run 12	Run 13	Run 14	Run 15	Run 16	Run 17	Run 18	Run 19	Total
Bacterial mock			5	7	2	19	4	3	3	2	2	3	12	1	13	4	14	105 (110)	14	213 (218)
Stool	8	15	6																	29
Negatives		(5)	(7)	(12)		(1)	(3)	(7)	(5)	(6)	(7)	(11)	(38)	(10)	(33)	(10)	1(27)	(13)	1 (27)	2(222)
Analysis																				
Mocks over time			●	●	●	●	●	●	●	●	●	●	●	^	●	●	●	*	●	117
Biological vs technical variation	●	●	●	●	●	●	●	●	●	●	●	●	●	^	●	●	●	*	●	146
Biomass																		●		105

^●^Included in the analysis

^^^Excluded due to only having a single mock sample in this run

*Only includes 10 undiluted mock samples from this run

### Quality control

Initial principal coordinates analysis (PCoA) revealed three distinct groups comprising the stool, mock community, and negative control samples (Additional file 1: Figure S1). Given the clear segregation of the negative controls, we identified and removed contaminant sequences based on their overrepresentation in negative control samples (Additional file 2: Figure S2a). Based on the distribution observed, we removed all sequence variants whose aggregate count in negative control samples exceeded 10% of the total count. Following this step, we examined the number of reads remaining for each sample and identified an inflection point at approximately 10,000 reads above which only two negative controls remained (Additional file 2: Figure S2b). We therefore chose to retain the samples with at least 10,942 reads for all subsequent analyses, leaving us with a total of 244 samples (2/222 (0.9%) negative control, 29/29 (100%) stool, 213/218 (97.7%) mock community) across 19 sequencing runs (Table 1).

### Intra- and inter-assay variation of a mock community over time

**Fig. 1:**
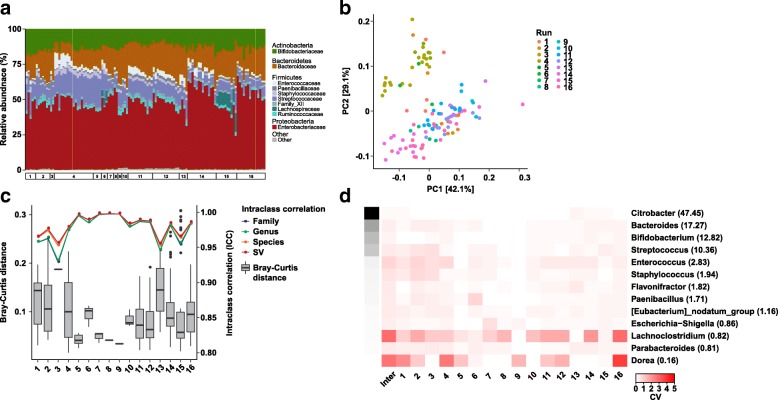
Bacterial mock community samples (*n* = 117) over time. **a** Taxonomic composition of bacterial mock community samples over the course approximately 2 years shown at the family level. Labeled boxes along the bottom denote individual sequencing runs. Only taxa with an average abundance of at least 1% are shown. **b** Principal coordinates analysis (PCoA) of bacterial mock community samples using Bray-Curtis distances. Numbers in brackets denote percent of variation explained. **c** Bray-Curtis distances (boxplots) and intraclass correlation coefficients (ICC) (line plots) stratified by sequencing run. ICC values are shown as means. **d** Heatmap of the coefficient of variation (CV) values for individual bacterial genera across sequencing runs. Grayscale cells on the left indicate mean relative abundances for each genus (also given as percentages in parentheses)

One hundred eighteen samples prepared from independent aliquots of a custom designed 33-strain mock community extracted and sequenced in 17 runs over the course of 2 years were selected for analysis. One run contained only a single mock sample and was therefore excluded, leaving a total of 117 samples across 16 runs. The inferred taxonomic compositions were highly consistent across all samples (Fig. 1a) even though they did not match the expected community composition (Additional file 3: Table S1). A total of 204 amplicon sequence variants (SV) were observed for the 33 strains, which is comparable to the error rates reported in recent benchmark studies [19, 20]. Clustering by run was observed (Fig. 1b) with a large proportion of variance explained (PERMANOVA *R*^2^ = 0.80, *p* < 0.001), although this measure is likely inflated by the inclusion of only a very tightly clustered set of samples. Measures of pairwise distance and intraclass correlation (ICC) stratified by sequencing run revealed generally high consistency across the sequencing runs (Fig. 1c, mean Bray-Curtis distance = 0.096, mean ICC = 0.98 at all family/genus/species/sequence variant levels). Of note, the overall ICC across all 16 runs ranged from 0.94 at the family level to 0.96 at the sequence variant level, which is in agreement with the PERMANOVA results suggesting a small run-to-run batch effect.

To quantify intra- and inter-assay variation, we calculated the coefficient of variation (CV) at the family, genus, species, and sequence variant levels (Fig. 1d and Additional file 4: Figure S3). Intra-assay variation was defined based on the variation identified within a sequencing run. Inter-assay variation looked at reproducibility of the same sample over multiple runs. Intra-assay coefficients of variation ranged from a mean of 8.7% (0.04–17.9%) for the most abundant genus, *Citrobacter*, with a relative abundance of 47.45, to a mean of 37.6% (1.6–87.6%) for *Enterococcus*, with a relative abundance of 2.83. One genus, *Lachnoclostridium*, had much greater variability with a mean intra-assay CV of 118.4% (not detected—201% with a relative abundance of 0.82). Mean CVs were remarkably consistent across taxonomic levels (family, genus, species, and SV) (Kruskal-Wallis *p* = 0.77, Additional file 4: Figure S3). For bacteria with a relative abundance < 1%, CVs were highly variable. Inter-assay variation ranged from 15.6% for *Citrobacter* to 80.4% for *Enterococcus* with *Enterococcus* having greater variability than some less abundant bacterial taxa. Overall, a significant negative correlation between CV and mean relative abundance at the family level (Fig. 1d, Additional file 4: Figure S3 and Additional file 5: Table S2) and other taxonomic levels was observed. Levey-Jennings plots further revealed the greatest run variance primarily among low-abundance bacterial families (Additional file 6: Figure S4).

### Does biological variation exceed technical variation?

**Fig. 2:**
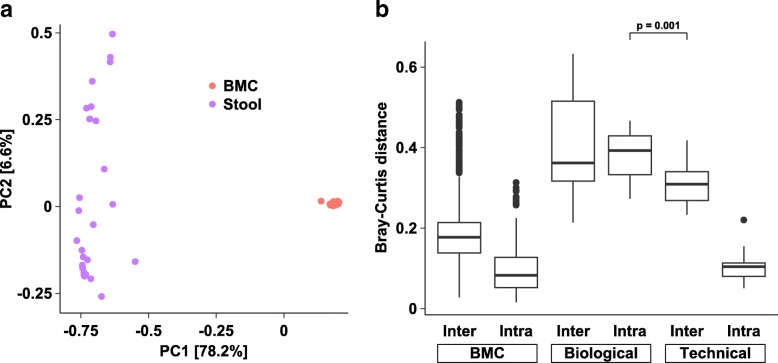
Overview of QC-filtered samples (*n* = 146). **a** Principal coordinates analysis (PCoA) on Bray-Curtis distances. Numbers in brackets denote percent of variation explained. **b** Boxplot of Bray-Curtis distances for bacterial mock and stool samples. Biological variation is shown between three samples from the same individual. Technical variation examines the same sample over multiple sequencing runs

A critical question in most microbiome studies is whether a biological signal can be distinguished from technical variation. To this end, we first jointly examined 146 quality control-filtered stool and undiluted bacterial mock samples using PCoA on Jensen-Shannon distances (JSD) (Fig. 2a). As expected, the tight clustering of the bacterial mock and stool samples was observed. Permutational multivariate analysis of variance (PERMANOVA) confirmed sample type as the dominant driver of variation (*R*^2^ = 0.65, *p* < 0.001), with sequencing run (*R*^2^ = 0.07, *p* < 0.001) also contributing significantly to overall variation. Intra- versus inter-assay Bray-Curtis distances for a single stool sample were 0.11 versus 0.31, whereas biological variation from the same subject 2 to 4 weeks apart was greater at 0.38 (Wilcoxon *p* = 0.001, Fig. 2b). Similar results were observed for other distance metrics (Additional file 7: Figure S5). Taken together, these findings suggest that even small biological effects (e.g., stool specimens from a single individual) can be readily distinguished from technical variation.

### How does input biomass affect technical variation?

Recent studies have highlighted the importance of absolute quantification of input DNA, particularly in studies where the samples are known to be of low biomass and susceptible to being overwhelmed by contamination [3, 9]. To quantitatively assess the impact of starting DNA amount on variability in microbial composition, a careful investigation of a dilution series of our bacterial mock community spanning the stock concentration to a thousandfold dilution was performed.

**Fig. 3:**
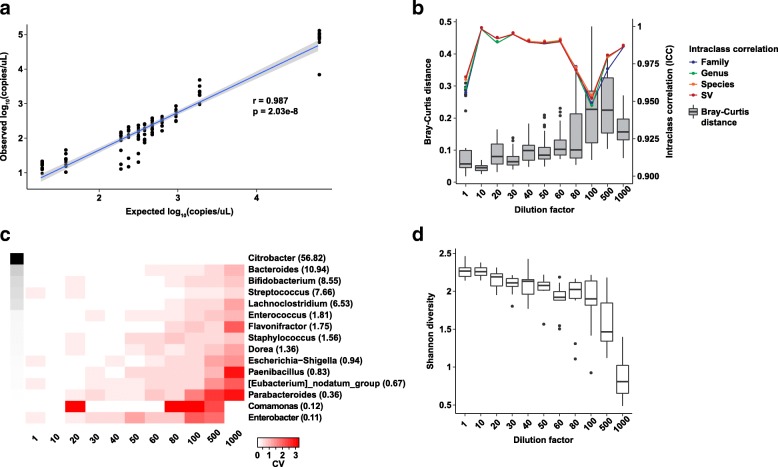
Variation as a function of input biomass. **a** Spearman correlation between expected 16S rRNA gene copies per microliter and calculated 16S rRNA gene copies per microliter. Values are log10-transformed. **b** Bray-Curtis distances (boxplots) and intraclass correlation coefficients (ICC) (line plots) stratified by dilution constant (e.g., 1:1 means stock, 1:1000 means diluted 1000-fold). ICC values are shown as means. **c** Heatmap of the coefficient of variation (CV) values for individual genera stratified by dilution constant. Grayscale cells on the left indicate mean relative abundances for each genus (also given as percentages in parentheses). **d** Shannon diversity as a function of dilution constant

Using 16S rRNA gene quantitative PCR (qPCR) as a measure of absolute abundance, the expected strong correlation between theoretical dilution constants and 16S rRNA gene copies was found (Fig. 3a Pearson *r* = 0.99). Notably, a small but consistent over-dilution effect in our series was observed; that is, the 16S rRNA gene copy numbers as measured by qPCR were monotonically lower than the expected copy numbers. This effect was somewhat ameliorated when the expected copy numbers were recalibrated to account for the sequential nature of the dilution series (e.g., 1:20 dilution was made from the 1:10 dilution). In the 16S rRNA gene sequencing data, we observed unexpected sequences classified as *Pseudomonas*, *Moraxella*, among others, most noticeably in the more dilute samples (Additional file 8: Figure S6a). Although the contaminant filtering steps removed this effect quite well (Additional file 8: Figure S6b), we still suggest that the 1:80 dilution series (approximately 94 copies of 16S rRNA gene/μL) may represent the lower limit of what can be accurately and robustly profiled without being significantly impacted by contamination. As expected, pairwise distances increased with more dilute samples (Fig. 3b) and approached the distances representative of biological variation (Fig. 2b) with the 1:100, 1:500, and 1:1000 dilutions. CV values at selected taxonomic levels (Fig. 3c, Additional file 9: Figure S7) also point to the unreliability of relative abundance estimates in samples past the 1:80 dilution set (~ 94 copies/μL). Finally, the expected decrease in alpha diversity estimates with more dilute samples (Fig. 3d) also suggests that the 1:80 dilution set be treated as the lower limit of what can be reliably measured.

### Predicting variation in 16S rRNA gene measurements

As the dilution coefficients here represent an artificial construct, an attempt was made to place estimates of variability in the context of absolute abundance measurements (e.g., 16S rRNA qPCR), so that they may be broadly applicable to microbiome studies.

**Fig. 4:**
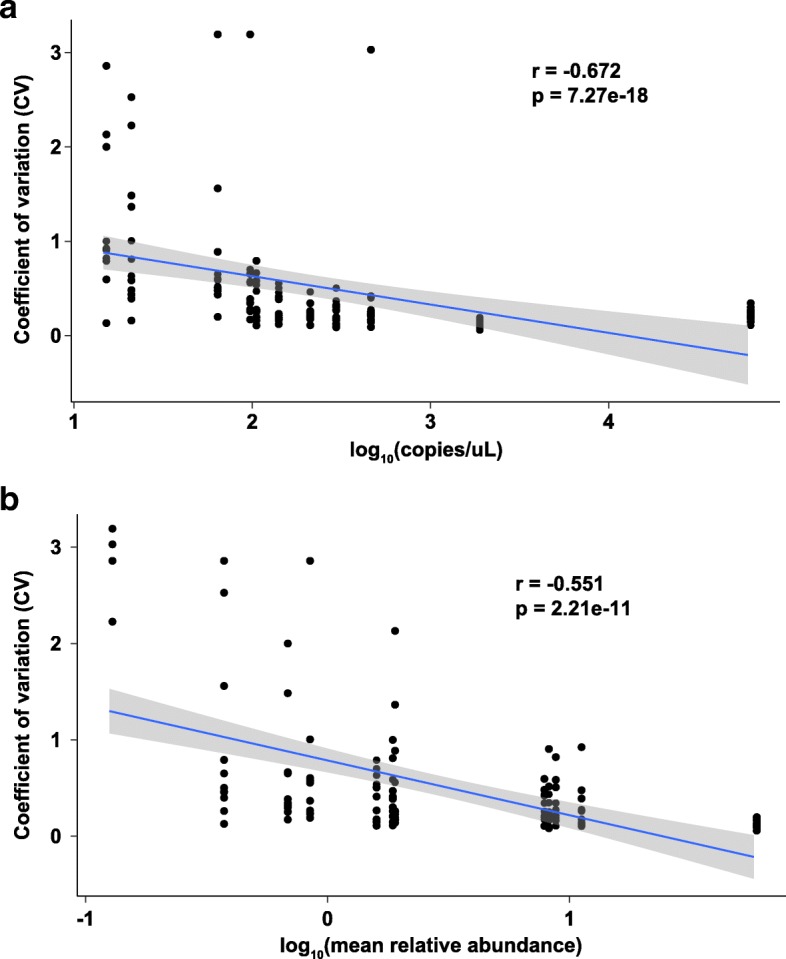
Modeling variation as a function of biomass and relative abundance. Spearman correlation between standard deviation in relative abundances (*y*-axis) and 16S rRNA gene copies/microliter (**a**) and mean relative abundance (**b**). All values are log10-transformed

As expected, CV values were negatively correlated with both absolute 16S rRNA gene copy number and mean relative abundance (Fig. 4, Spearman rho = − 0.67 and − 0.55, respectively). Multivariate linear regression revealed the same effect and further allowed a predictive model of the expected variation in microbiome measurements for given input biomass and relative abundance values to be created (Table 2, Additional file 10: Table S3).

**Table 2:** Linear regression modeling of variation versus input biomass and mean relative abundance. Summary of the linear regression model (a) and predicted variation values for a subset of 16S rRNA gene copies/microliter and mean relative abundance values (b)

a. Model	Estimate	Standard error	*t* value	*p* value		
(Intercept)	1.43238	0.12556	11.408	< 2.2E−16		
log10 copies/microliter	− 0.2816	0.04905	− 5.741	6.93E−8		
log10 mean relative abundance	− 0.54936	0.06927	− 7.931	1.13E−12		
Residual standard error	0.5196					
Multiple *R*-squared	0.4496					
Adjusted *R*-squared	0.4407					
*F* statistic	50.25 on DF (2123)					
b. Prediction		Mean relative abundance (%)				
	Copies/microliter	1	5	10	25	50
Low biomass	10	0.5723	1.7313	2.7888	5.2376	8.4370
Medium biomass	1000	0.2865	0.8668	1.3963	2.6224	4.2242
High biomass	100,000	0.1435	0.4340	0.6991	1.3130	2.1150

## Discussion

In this study, we characterized intra- and inter-assay variation, providing coefficients of variation, in targeted 16S rRNA gene sequencing studies in ideal experimental models and biological specimens. Additionally, the effect of low-biomass input on expected variance was explored, and a prediction model was created. These results provide context for the amount of technical variation to be expected over the course of long-running microbiome studies and may be useful in both study design and in assessing the validity of minute compositional differences in other datasets.

As discussed in the literature, the inclusion and analysis of negative control samples is critical in microbiome studies [9]. Although the filtering and quality control steps described here appear ad hoc, they are strongly motivated by the empiric observations in the data. Recent approaches to identify and remove contaminant sequences from microbiome sequencing data utilize frequency and prevalence of sequences in low-biomass samples and negative controls in a manner that may be less ad hoc than the approach described herein [5, 21]. Nevertheless, we wish to highlight the importance of the thoughtful application of data from negative controls to remove as much unwanted variance as possible from microbiome datasets.

Although mock communities are becoming much better characterized and applied to microbiome research [22], little has been done to explicitly follow these over time. We demonstrated with our repeated mock controls over a 2-year time frame that run-to-run variation may be limited to a fraction of the overall variation observed as long as procedures for extraction, amplification, and sequencing remain identical. We recommend following the known bacterial species in mock control samples using Levey-Jennings plots to help identify runs that may represent outliers in terms of bacterial composition. While we found good precision in amplification of our mock control community, accuracy was marginal. Accuracy likely reflects the limitations of the current databases, quantification of input, variability in extraction, and PCR amplification. This is consistent with previous studies that found higher precision than accuracy in microbiome profiling data [9, 22]. Of note, the expected taxonomic proportions for the mock community are based on whole-cell estimates and likely do not reflect post-extraction DNA content. We intentionally used a whole-cell mock control as we believe it is necessary to control for variation in the DNA extraction step; the debate between whole-cell versus DNA mock communities is a topic of much current interest [23].

When examining individual bacterial taxa, *Bifidobacterium* showed high consistency in relative abundance measurements. This robustness may help explain why small changes in *Bifidobacterium* abundances can be reliably detected [24, 25]. Interestingly, the trifecta of *Streptococcus*, *Enterococcus*, and *Staphylococcus* tended to be more variable, at least in a subset of the sequencing runs. It remains unclear whether this was due to technical variation in extraction and amplification. Furthermore, certain species may present specific difficulties and may be subject to even greater variability, such as *Dorea formicigenerans* in our mock community. Thus, while inter-assay coefficients of variation may be as low as 15.6% and usually fall under 80%, these values must be interpreted with caution in the context of the specific bacteria of interest.

Further concerns for technical variation outweighing biological variation have made interpretation of human microbiome studies difficult. Many studies have shown now that storage, extraction technique, sequencing platform, and analysis pipeline can all have effects on the results of a study. Further, a recent study of mock controls demonstrates that most of the technical variation occurred with extraction and amplification and not during the sequencing itself [10]. That being said, we found that under well-controlled experimental conditions, repeat samples showed little intra- or inter-assay variation, especially in taxa with high relative abundance. Inter-assay coefficients of variation generally exceeded intra-assay variation, although both sets of values were within the ranges reported in previous studies of microbiome stability [26–28]. Although accuracy potentially may not be great, the ability to replicate results with these standard methods with precision is there and argues for biological variation to be the key contributor of these types of studies.

The complexities associated with low biomass studies have been documented [9, 14]. Salter et al. elegantly showed the significant impact of kit contaminants on low-biomass samples both by a serial dilution study and re-analysis of a nasopharyngeal study. This is highlighted in recent reports of a commensal placenta microbiome followed by conflicting studies that revealed no evidence for a placental microbiome distinct from contamination controls [2, 3, 29]. In our dilution study of a known bacterial community, both absolute 16S rRNA gene copy number and mean relative abundance affected the variance in microbiome measurements. As the coefficient of variation is a standardized measurement of dispersion, the implication of this relationship is that additional variation is present in amplicon-based microbiome data beyond that derived from the underlying bacterial counts. Indeed, a number of recent studies have suggested that modeling of overdispersion as well as zero inflation is critical to the accurate interpretation of microbiome sequencing data [30, 31]. We further propose that these methods should incorporate the effect of input biomass as this may improve the ability to control false discoveries particularly for lower biomass samples.

Our study has a number of limitations. This is primarily an investigation at the intra- and inter-assay variation of samples being processed through a standardized pipeline and therefore may not be applicable to studies using other procedures. Regardless, because of the large number of ongoing studies in our microbiome-sequencing laboratory, we have a large number of repeated samples for intra- and inter-assay variation as well as positive and negative controls. The mock community we use is of relatively low complexity, and thus, the number of samples limits measures of biologic variation. Furthermore, the repeat stool samples from a single donor taken at 2-week intervals likely represent minimal biological variation given the relatively short time frame of their collection and the limited number of samples. Indeed, the measured inter-assay technical variation was surprisingly close to the biological intra- and inter-assay variation. We hypothesize that this is due to the limited number of comparisons made in this study and would caution that future studies to explore biological variation encompass a far greater diversity of specimens. Given the large sample numbers and commonly used sequencing protocol, we feel that this study provides a real-world experience and helps establish a baseline for the amount of biological and technical variation one should expect in a microbiome study.

## Conclusions

In this study, we characterized the variation that can be expected from well-controlled microbiome experiments to help establish a baseline so that future studies may be interpreted more accurately. Under well-controlled conditions, intra- and inter-assay variation should have little impact on the validity of the results. We further demonstrated that minimal biological variation (e.g., three repeat stool samples from the same donor) exceeds technical variation. Lastly, we show that the relative abundance has a stronger impact on variability than input biomass. In quantifying the effect of biomass and relative abundance on compositional variability, we provide a tool that will allow future microbiome studies to a priori define the expected variance.

## Methods

### Overall study design

A total of 469 samples from 19 separate sequencing runs spanning 2.5 years were analyzed in aggregate. Independent aliquots of a whole-cell bacterial mock community totaling 118 samples were included on 17 runs. One run (run 14) only contained a single mock sample which was excluded from further analysis, leaving a total of 117 samples across 16 runs. A prospective dilution study of the same mock community was conducted on a single run (run 18). Dilutions of 1:10, 1:20, 1:30, 1:40, 1:50, 1:60, 1:80, 1:100, 1:500, and 1:1000 bacterial mock community to sterile water were prepared and stored at − 80 °C until extraction. A total of 29 libraries prepared from three distinct stool specimens from the same donor taken at 1-week intervals were included on three runs. Negative controls were included on 17 of the 19 runs. Further details are provided below.

### Positive and negative controls

Our bacterial mock community comprises 33 clinical and ATCC strains (Additional file 3: Table S1). Colonies from sheep blood agar plates were resuspended in sterile normal saline. These were standardized to 1.0 MacFarland optic density based on nephelometer reading. Additional bacteria were added straight from glycerol stock as supplied by BEI Resources (Manassas, VA).

Aliquots of this bacterial mock community were prepared and stored in sterile water at − 80 °C until use. Negative controls included DNA extraction controls (e.g., reagents from DNA extraction kit) as well as PCR blanks using PCR-grade water with no DNA template.

### DNA extraction

After addition of RLT+ lysis buffer from the AllPrep DNA/RNA Mini Kit (Qiagen, Hilden, Germany), samples were transferred to Lysing Matrix E beads (MP Biomedicals, California, USA) and heated at 37 °C for 10 min with shaking at 700–800 rpm. This was followed by bead beating on a TissueLyser II (Qiagen) system. The supernatant was then removed from the centrifuged samples and placed on an automated QIAcube (Qiagen) workflow system. DNA was then extracted using the AllPrep DNA/RNA Mini Kit (Qiagen) in accordance with the manufacturer’s protocols. Extracted DNA was stored in elution buffer at − 80 °C.

### Library preparation

The 16S rDNA was amplified in triplicate and barcoded using a previously published protocol (*26*). Briefly, 1 μL of the extracted DNA was amplified using primers complementary to the V4 (515F/806R) region of the 16S rRNA gene in a single-step PCR reaction. Illumina (San Diego, CA, USA) flow cell adapter sequences and a 12-bp barcode were incorporated into the PCR primers, yielding a fully Illumina-compatible sequencing library. DNA amplicon purity and concentration was quantified on a 2100 BioAnalyzer (Agilent Technologies, Santa Clara, California, USA) and Qubit 3.0 Fluorometer (Thermo Fisher Scientific, Waltham, MA, USA).

### Sequencing

We followed the detailed sequencing protocol as presented previously by Caporaso et al. [32]. Briefly, unless stated otherwise, we diluted each sample to 2 nM. Amplicons were then denatured and loaded onto a MiSeq desktop sequencer (Illumina, San Diego, CA, USA) using 2 × 150 bp v2 chemistry. After cluster formation, the amplicons were sequenced with custom primers complementary to the 515F/806R amplification primers, thereby avoiding sequencing of the constant regions at the ends of the target amplicon. The barcode was read using a third sequencing primer in an additional set of cycles.

### Quantitative PCR

The positive mock bacterial control dilution series was further analyzed by quantitative PCR. Degenerate primers were modified from the original 515F-806R primer pair by removal of the linker, pad, barcode, and adapter sequences [32]. (Primer sequences—forward: GTGYCAGCMGCCGCGGTAA; reverse: GGACTACNVGGGTWTCTAAT) Real-time quantitative PCR was done using PerfeCTa SYBR Green FastMix ROX (Quanta Bio, Catalog 95073-012) using a 384-well format on an ABI 7900HT machine. All samples were run in triplicate wells. Run setup was based on Quanta Bio’s product manual using standard cycling (95 °C for 3 min, followed by 40 cycles of 95 °C for 15 s, 50°for 60 s). We used 5 μL of template per reaction for a final reaction volume of 20 μL per sample. A plasmid-encoded single-copy 16S rRNA gene standard was used at the following concentrations (copies/5 μL): 0, 10^2^, 10^3^, 10^4^, 10^5^, 10^6^, and 10^7^.

### Data analysis and statistics

Sequences were demultiplexed with Golay error correction using QIIME 1.9.1 [32]. Divisive amplicon denoising algorithm version 2 (DADA2) was used for error correction, exact sequence inference, and chimera removal [33]. All statistical analyses, including the calculation of alpha and beta diversity metrics and taxonomic compositions, were performed using the “phyloseq” package in the R software environment (version 3.3.2) [34].

Removal of contaminant sequence variants was performed by a simple “contaminant score” *S*_*i*_:

$$ {S}_i=\frac{\sum \limits_j{c}_{ij}}{\sum {c}_i} $$

For sequence *i*, *j* being the set of negative control samples, and *c*_*ij*_ being the read count of sequence *i* in sample *j*. This score ranges from 0 for sequences that are only observed in “true” samples to 1 for sequences that are only observed in negative controls. Intermediate values are interpreted as a measurement of the likelihood that a given sequence variant was derived from negative controls (i.e., contamination) as opposed to being truly present in a sample of interest. We used a threshold of 0.1 to identify and remove contaminant SVs prior to all further analysis. Following removal of contaminant SVs, a rarefaction depth of 10,942 reads was selected based on the empirically observed distribution of read counts. The rarefied SV table was used for analysis of alpha diversity, and relative abundances were used for all other analyses.

Additional statistical analyses were performed using R statistical software version 3.3.1. Nonparametric Kruskal-Wallis and Wilcoxon tests were used as described in the text. Mean relative abundance when used in the text refers to the mean of per-sample relative abundance values for a given taxon.

### Analysis of bacterial mock community over time

A total of 118 samples over 17 sequencing runs were initially selected for this analysis. Of note, one run (run 14) only contained a single mock sample and was therefore excluded from the analysis. Additionally, ten undiluted mock samples from the dilution study run (run 18 in Table 1) were included in the *n* = 117 final set. Intra-assay measures of variability (e.g., stratified by run) were computed by taking subsets of data for each sequencing run and computing the associated statistic, whereas the inter-assay variability was computed using the entire data matrix of 117 samples. The intraclass correlation coefficient (ICC), a commonly used statistic of measurement reproducibility, was computed using the “irr” R package (version 0.84) using a one-way model.

### Analysis of biological versus technical variation

A total of 147 samples over 19 sequencing runs were initially selected for this analysis. One run (run 14) only contained a single mock sample and was therefore excluded, leaving a total of 146 samples across 18 runs for analysis. Pairwise distances were first computed for all 146 samples, and then the appropriate elements were extracted for each comparison group.

### Analysis of input biomass and variation

A total of 105 samples from a dilution series sequenced in a single run (run 18) were used for this analysis. Multivariate linear regression was performed using the “stats” base R package and a model of *coefficient of variation ~ log10(qPCR copies per μL) + log10(mean relative abundance)* for each taxa of interest.

## Additional files

Additional file 1: **Figure S1.** Principal coordinates analysis (PCoA) on Bray-Curtis distances for all samples (*n* = 469), prior to contaminant sequence variant (SV) removal and filtering. (PDF 599 kb)

Additional file 2: **Figure S2.** Removal of contaminant sequence variants (SVs). a) Frequency plot of percentage of reads derived from negative controls for each SV. b) Read counts for each sample after removal of contaminant SVs. Horizontal dotted line at approximately 10,000 reads is the rarefaction threshold (10942). (PDF 112 kb)

Additional file 3: **Table S1.** Composition of the bacterial mock community. (DOCX 17 kb)

Additional file 4: **Figure S3.** Heatmaps of coefficient of variation (CV) values for each taxon across sequencing runs, at the family (a), species (b), and sequence variant (c) levels. Greyscale cells on the left indicate mean relative abundances for each taxon (also given as percentages in parentheses). (PDF 692 kb)

Additional file 5: **Table S2.** Coefficient of variation (CV) values for individual bacteria taxa in the bacterial mock community samples measured across 16 sequencing runs. (XLSX 29 kb)

Additional file 6: **Figure S4.** Levey-Jennings plots for bacterial genera over the course of 16 sequencing runs (*x*-axis, sorted chronologically from left to right). Mean relative abundance (solid line), one standard deviation (dashed line), and two standard deviations (dotted line) are indicated. Samples in red represent observations more than two standard deviations from the mean. (PDF 972 kb)

Additional file 7: **Figure S5.** Boxplots of Jensen-Shannon (a) and Jaccard (b) distances for bacterial mock and stool samples. (PDF 257 kb)

Additional file 8: **Figure S6.** Taxonomic composition of bacterial mock community samples pre-filtering (a) and post-filtering (b). Compositions are sorted by dilution constant and shown at the family level. Shading along bottom indicates less (darker) and more (lighter) dilution. Only taxa with an average abundance of at least 1% are shown. (PDF 253 kb)

Additional file 9: **Figure S7.** Heatmaps of coefficient of variation (CV) values for each taxon by dilution constant, at the family (a), species (b), and sequence variant (c) levels. Greyscale cells on the left indicate mean relative abundances for each taxon (also given as percentages in parentheses). (PDF 484 kb)

Additional file 10: **Table S3.** Predictive models of the expected variation in microbiome measurements for given input biomass and relative abundance values at the family, species, and sequence variant level. (XLSX 15 kb)

## Abbreviations

CV: Coefficient of variation
ICC: Intraclass correlation coefficient
JSD: Jensen-Shannon distances
PCoA: Principal coordinates analysis
PERMANOVA: Permutational multivariate analysis of variance
qPCR: Quantitative PCR
SV: Sequence variant

## References

[1] Knight R, Callewaert C, Marotz C, Hyde ER, Debelius JW, McDonald D, Sogin ML (2017). The microbiome and human biology. Annu Rev Genomics Hum Genet.

[2] Perez-Munoz ME, Arrieta MC, Ramer-Tait AE, Walter J (2017). A critical assessment of the “sterile womb” and “in utero colonization” hypotheses: implications for research on the pioneer infant microbiome. Microbiome.

[3] Lauder AP, Roche AM, Sherrill-Mix S, Bailey A, Laughlin AL, Bittinger K, Leite R, Elovitz MA, Parry S, Bushman FD (2016). Comparison of placenta samples with contamination controls does not provide evidence for a distinct placenta microbiota. Microbiome.

[4] Brooks B, Firek BA, Miller CS, Sharon I, Thomas BC, Baker R, Morowitz MJ, Banfield JF (2014). Microbes in the neonatal intensive care unit resemble those found in the gut of premature infants. Microbiome.

[5] Minich JJ, Zhu Q, Janssen S, Hendrickson R, Amir A, Vetter R, Hyde J, Doty MM, Stillwell K, Benardini J, et al. KatharoSeq enables high-throughput microbiome analysis from low-biomass samples. mSystems. 2018;3(3) 10.1128/mSystems.00218-17. 10.1128/mSystems.00218-17 PMC5864415 29577086

[6] Raising standards in microbiome research. Nature Microbiology. 2016;1(7):16112. 10.1038/nmicrobiol.2016.112. 10.1038/nmicrobiol.2016.112 27572984

[7] Clooney AG, Fouhy F, Sleator RD, A OD, Stanton C, Cotter PD, Claesson MJ (2016). Comparing apples and oranges?: next generation sequencing and its impact on microbiome analysis. PLoS One.

[8] Sinha R, Abnet CC, White O, Knight R, Huttenhower C (2015). The microbiome quality control project: baseline study design and future directions. Genome Biol.

[9] Kim D, Hofstaedter CE, Zhao C, Mattei L, Tanes C, Clarke E, Lauder A, Sherrill-Mix S, Chehoud C, Kelsen J (2017). Optimizing methods and dodging pitfalls in microbiome research. Microbiome.

[10] Brooks JP, Edwards DJ, Harwich MD, Rivera MC, Fettweis JM, Serrano MG, Reris RA, Sheth NU, Huang B, Girerd P (2015). The truth about metagenomics: quantifying and counteracting bias in 16S rRNA studies. BMC Microbiol.

[11] Glassing A, Dowd SE, Galandiuk S, Davis B, Chiodini RJ (2016). Inherent bacterial DNA contamination of extraction and sequencing reagents may affect interpretation of microbiota in low bacterial biomass samples. Gut Pathog.

[12] Gerasimidis K, Bertz M, Quince C, Brunner K, Bruce A, Combet E, Calus S, Loman N, Ijaz UZ. The effect of DNA extraction methodology on gut microbiota research applications. BMC Res Notes. 2016;9(1) 10.1186/s13104-016-2171-7. 10.1186/s13104-016-2171-7 PMC4960752 27456340

[13] Kennedy NA, Walker AW, Berry SH, Duncan SH, Farquarson FM, Louis P, Thomson JM, Satsangi J, Flint HJ, Parkhill J (2014). The impact of different DNA extraction kits and laboratories upon the assessment of human gut microbiota composition by 16S rRNA gene sequencing. PLoS One.

[14] Salter SJ, Cox MJ, Turek EM, Calus ST, Cookson WO, Moffatt MF, Turner P, Parkhill J, Loman NJ, Walker AW (2014). Reagent and laboratory contamination can critically impact sequence-based microbiome analyses. BMC Biol.

[15] Wagner Mackenzie B, Waite DW, Taylor MW (2015). Evaluating variation in human gut microbiota profiles due to DNA extraction method and inter-subject differences. Front Microbiol.

[16] Jones MB, Highlander SK, Anderson EL, Li W, Dayrit M, Klitgord N, Fabani MM, Seguritan V, Green J, Pride DT (2015). Library preparation methodology can influence genomic and functional predictions in human microbiome research. Proc Natl Acad Sci U S A.

[17] D’Amore R, Ijaz UZ, Schirmer M, Kenny JG, Gregory R, Darby AC, Shakya M, Podar M, Quince C, Hall N (2016). A comprehensive benchmarking study of protocols and sequencing platforms for 16S rRNA community profiling. BMC Genomics.

[18] Choo JM, Leong LE, Rogers GB (2015). Sample storage conditions significantly influence faecal microbiome profiles. Sci Rep.

[19] DADA2 and the State of the Art [http://benjjneb.github.io/dada2/SotA.html].

[20] Nearing JT, Douglas GM, Comeau AM, Langille MGI (2018). Denoising the denoisers: an independent evaluation of microbiome sequence error-correction methods. PeerJ.

[21] Davis N, Proctor D, Holmes S, Relman DA, Callahan BJ: Simple statistical identification and removal of contaminant sequences in marker-gene and metagenomics data. bioRxiv 2017. 10.1101/221499. 10.1186/s40168-018-0605-2 PMC6298009 30558668

[22] Singer E, Andreopoulos B, Bowers RM, Lee J, Deshpande S, Chiniquy J, Ciobanu D, Klenk HP, Zane M, Daum C (2016). Next generation sequencing data of a defined microbial mock community. Scientific data.

[23] Pollock J, Glendinning L, Wisedchanwet T, Watson M. The madness of microbiome: attempting to find consensus “best practice” for 16S microbiome studies. Appl Environ Microbiol. 2018;84(7) 10.1128/AEM.02627-17. Print 2018 Apr 1. 10.1128/AEM.02627-17 PMC5861821 29427429

[24] Bonder MJ, Kurilshikov A, Tigchelaar EF, Mujagic Z, Imhann F, Vila AV, Deelen P, Vatanen T, Schirmer M, Smeekens SP (2016). The effect of host genetics on the gut microbiome. Nat Genet.

[25] Sivan A, Corrales L, Hubert N, Williams JB, Aquino-Michaels K, Earley ZM, Benyamin FW, Lei YM, Jabri B, Alegre ML (2015). Commensal Bifidobacterium promotes antitumor immunity and facilitates anti-PD-L1 efficacy. Science.

[26] Shaw AG, Sim K, Powell E, Cornwell E, Cramer T, McClure ZE, Li MS, Kroll JS (2016). Latitude in sample handling and storage for infant faecal microbiota studies: the elephant in the room?. Microbiome.

[27] Schloss PD, Schubert AM, Zackular JP, Iverson KD, Young VB, Petrosino JF (2012). Stabilization of the murine gut microbiome following weaning. Gut Microbes.

[28] Utter DR, Mark Welch JL, Borisy GG (2016). Individuality, stability, and variability of the plaque microbiome. Front Microbiol.

[29] Aagaard K, Ma J, Antony KM, Ganu R, Petrosino J, Versalovic J (2014). The placenta harbors a unique microbiome. Sci Transl Med.

[30] Weiss S, Xu ZZ, Peddada S, Amir A, Bittinger K, Gonzalez A, Lozupone C, Zaneveld JR, Vázquez-Baeza Y, Birmingham A (2017). Normalization and microbial differential abundance strategies depend upon data characteristics. Microbiome.

[31] Thorsen J, Brejnrod A, Mortensen M, Rasmussen MA, Stokholm J, Al-Soud WA, Sorensen S, Bisgaard H, Waage J (2016). Large-scale benchmarking reveals false discoveries and count transformation sensitivity in 16S rRNA gene amplicon data analysis methods used in microbiome studies. Microbiome.

[32] Caporaso JG, Lauber CL, Walters WA, Berg-Lyons D, Huntley J, Fierer N, Owens SM, Betley J, Fraser L, Bauer M (2012). Ultra-high-throughput microbial community analysis on the Illumina HiSeq and MiSeq platforms. ISME J.

[33] Callahan BJ, McMurdie PJ, Rosen MJ, Han AW, Johnson AJ, Holmes SP (2016). DADA2: high-resolution sample inference from Illumina amplicon data. Nat Methods.

[34] McMurdie PJ, Holmes S (2013). phyloseq: an R package for reproducible interactive analysis and graphics of microbiome census data. PLoS One.

