# Supplementary figures and images for: Quantification of variation and the impact of biomass in targeted 16S rRNA gene sequencing studies
Source: Microbiome. 2018 Sep 10;6:155. doi: 10.1186/s40168-018-0543-z (PMC6131952; doi:10.1186/s40168-018-0543-z)

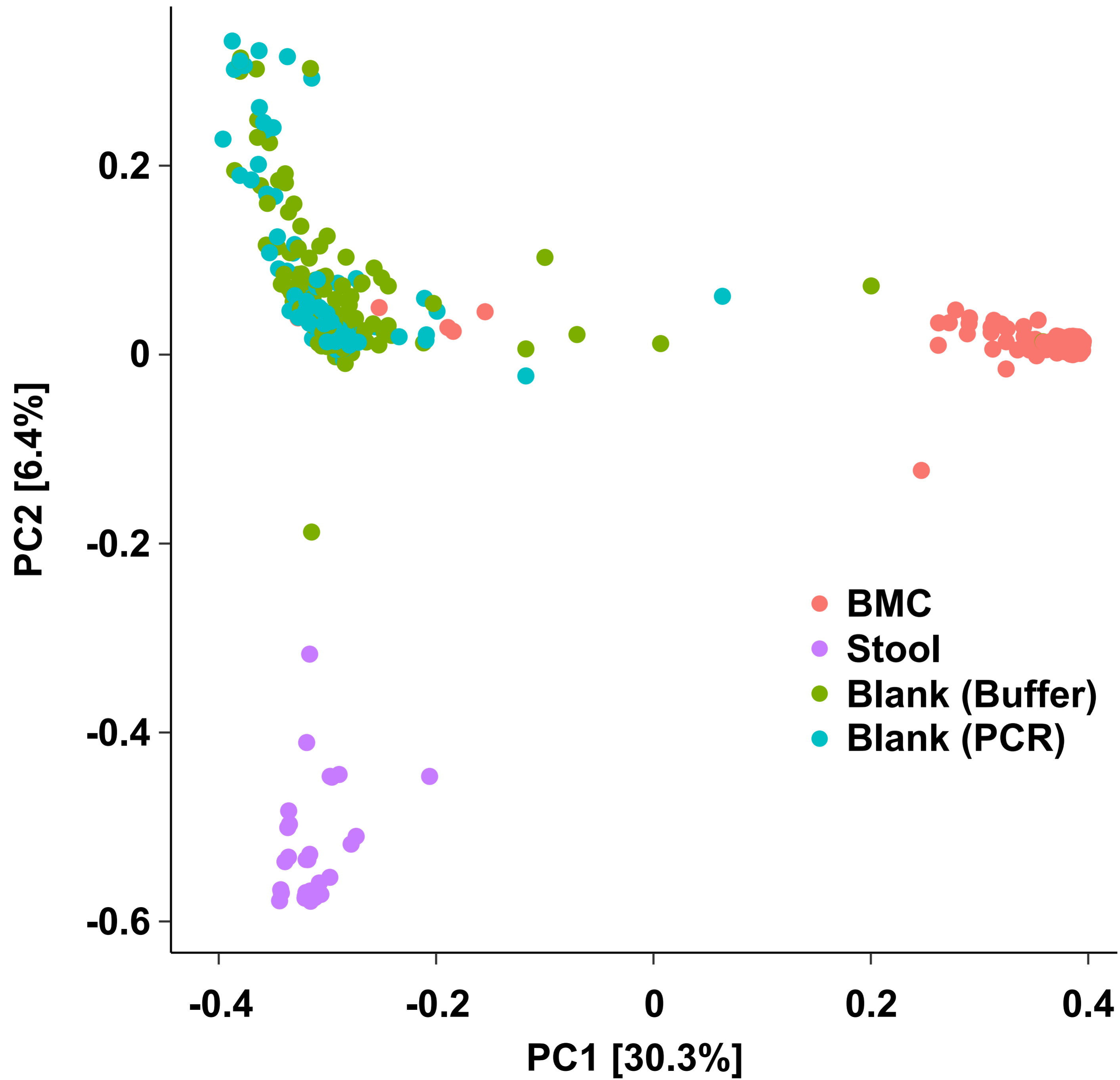

Supplement: Supplementary file 1 — Figure S1. Principal coordinates analysis (PCoA) on Bray-Curtis distances for all samples (n = 469), prior to contaminant sequence variant (SV) removal and filtering. (PDF 599 kb) [file 40168_2018_543_MOESM1_ESM.pdf]

a.

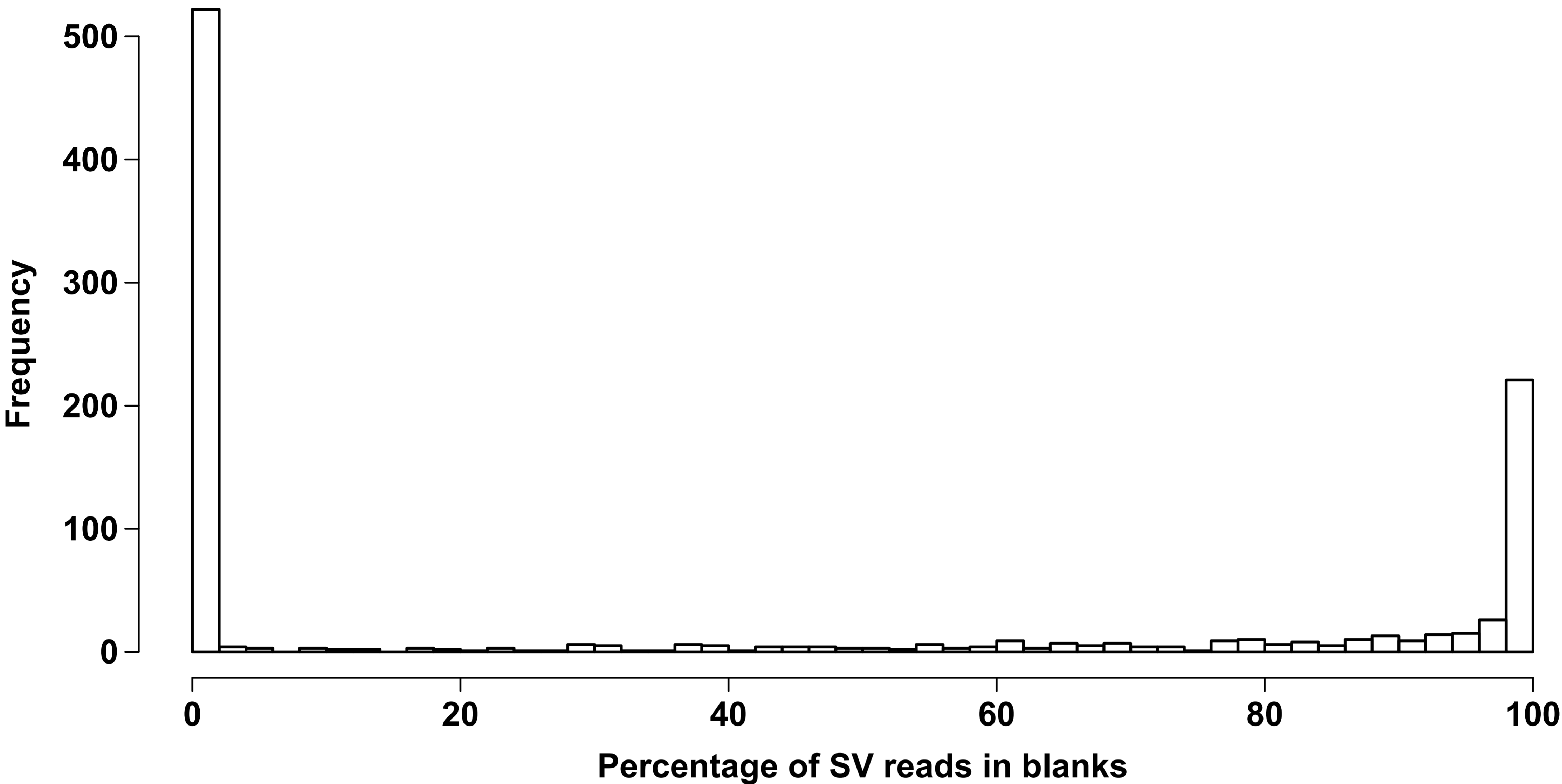

b.

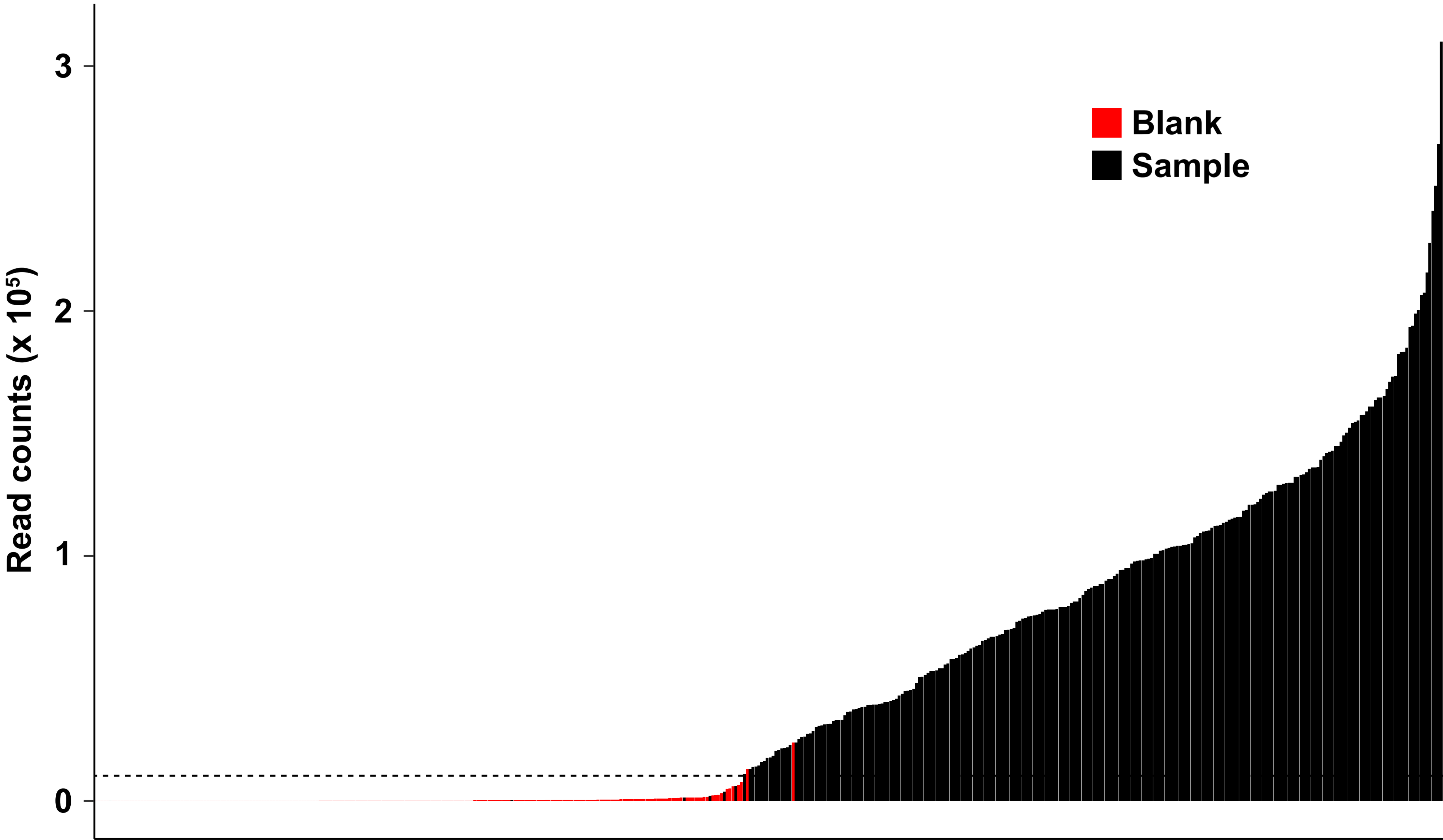

Supplement: Supplementary file 2 — Figure S2. Removal of contaminant sequence variants (SVs). a) Frequency plot of percentage of reads derived from negative controls for each SV. b) Read counts for each sample after removal of contaminant SVs. Horizontal dotted line at approximately 10,000 reads is the rarefaction threshold (10942). (PDF 112 kb) [file 40168_2018_543_MOESM2_ESM.pdf]

a.

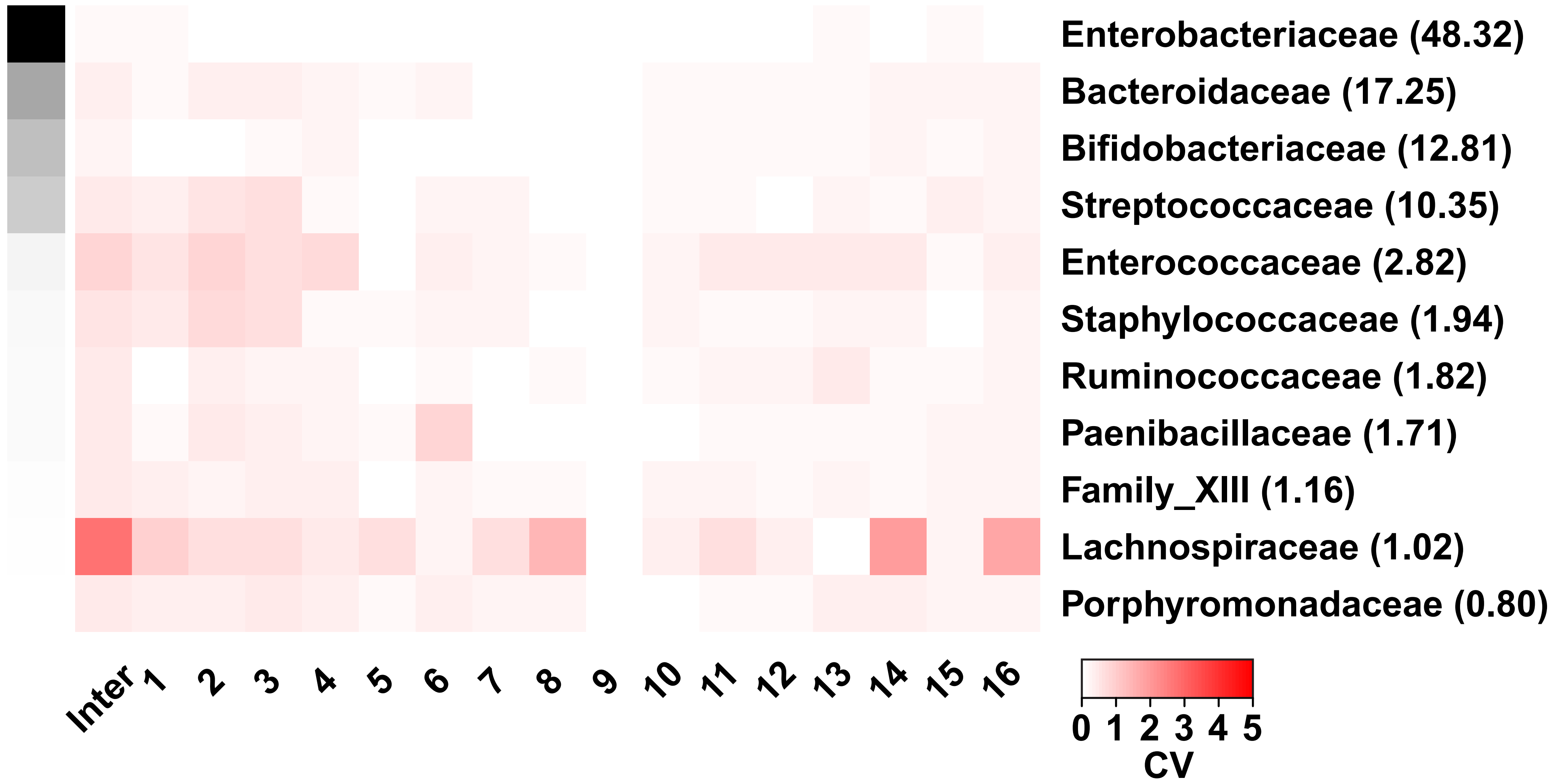

b.

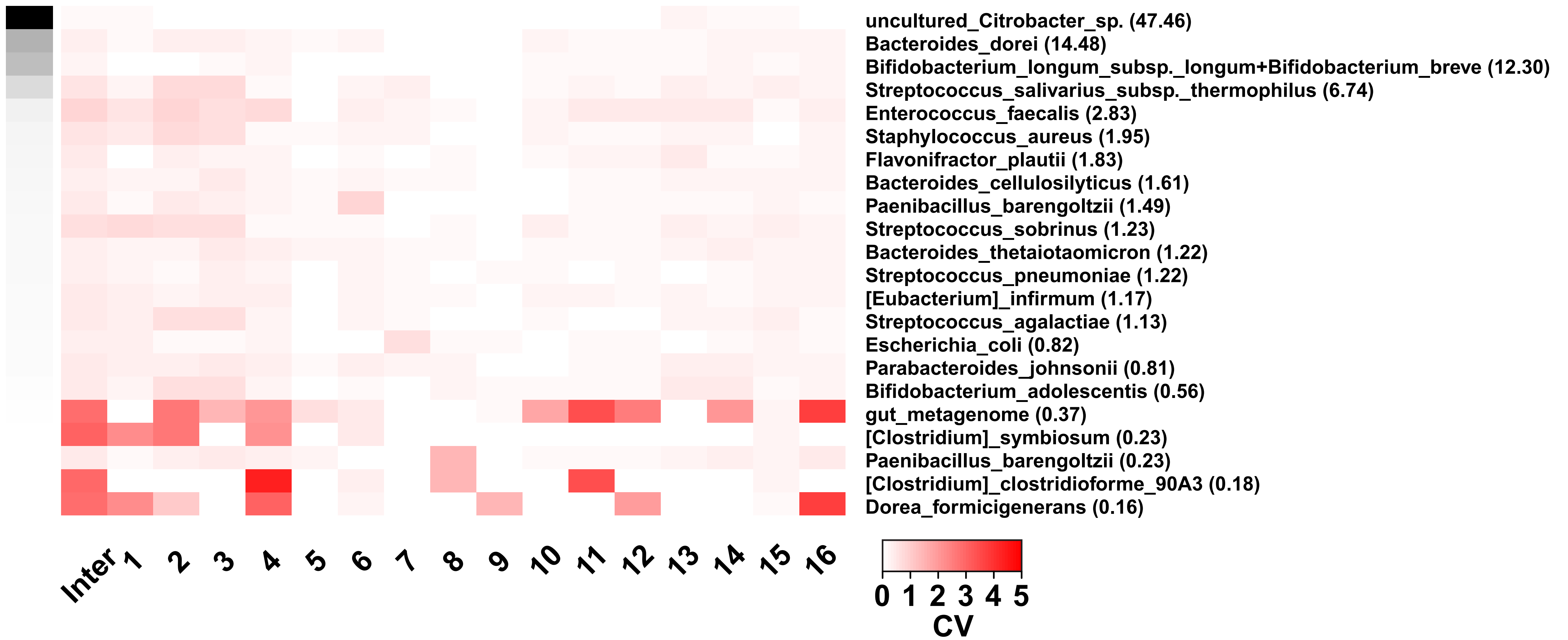

c.

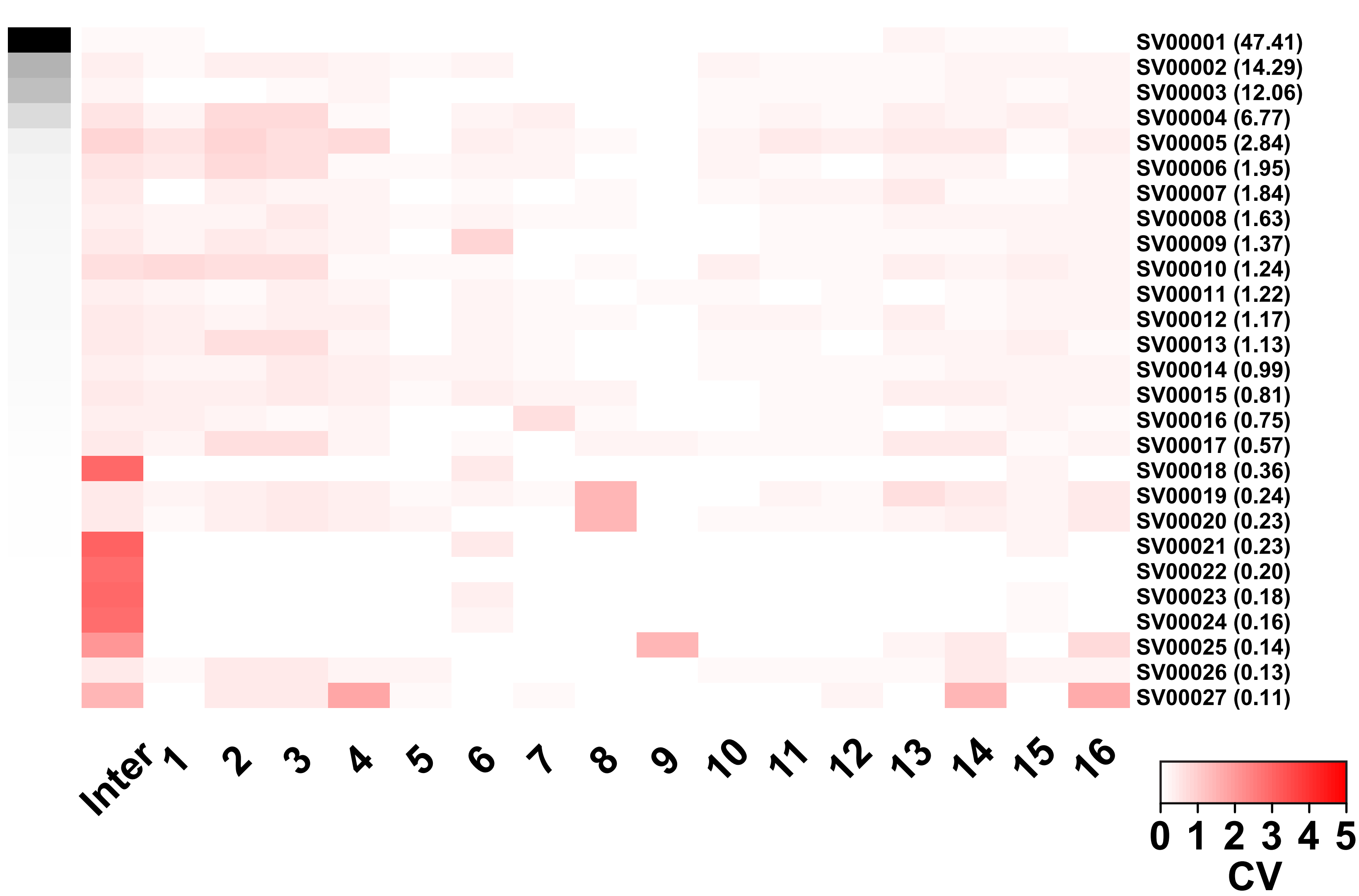

Supplement: Supplementary file 4 — Figure S3. Heatmaps of coefficient of variation (CV) values for each taxon across sequencing runs, at the family (a), species (b), and sequence variant (c) levels. Greyscale cells on the left indicate mean relative abundances for each taxon (also given as percentages in parentheses). (PDF 692 kb) [file 40168_2018_543_MOESM4_ESM.pdf]

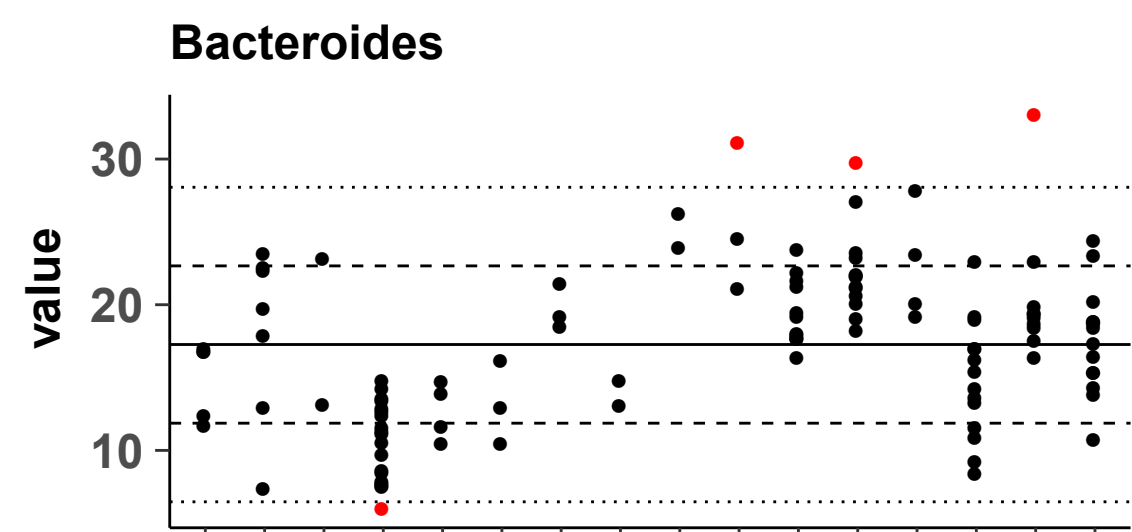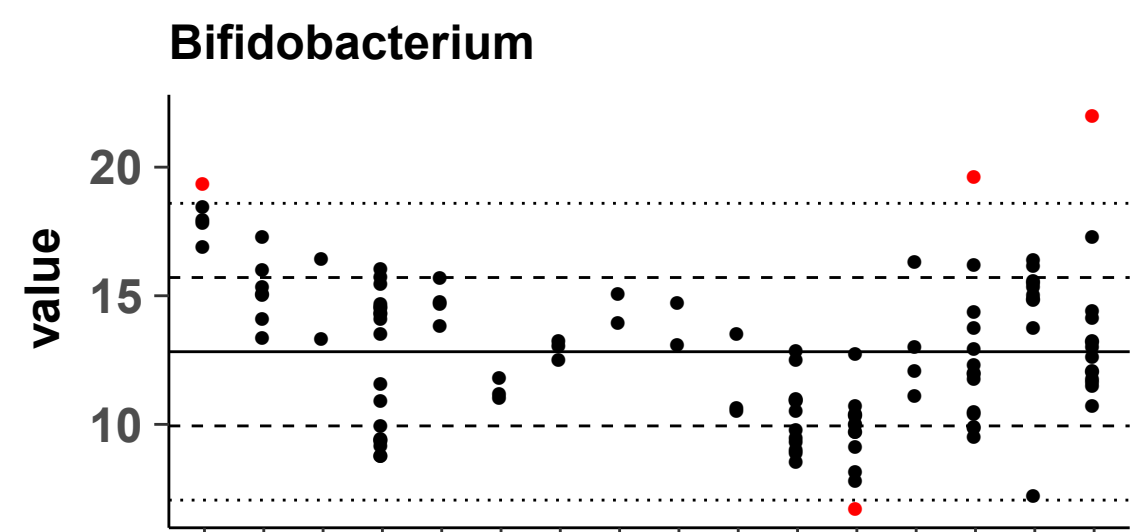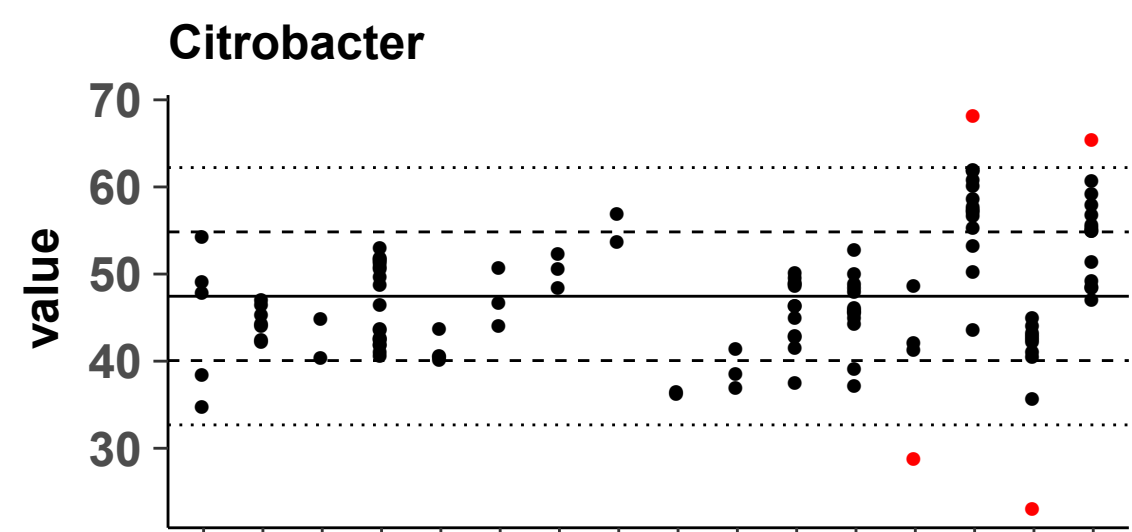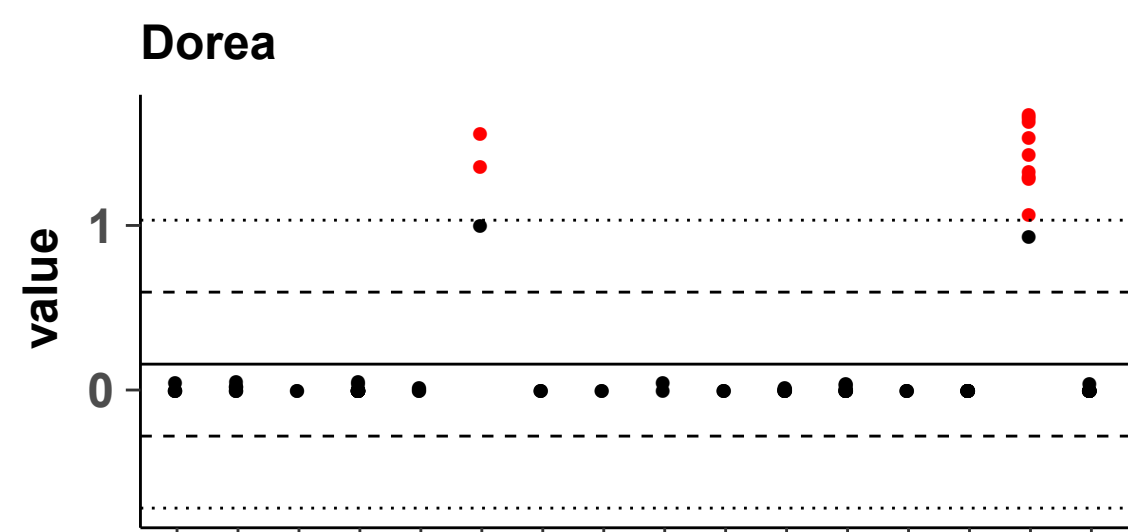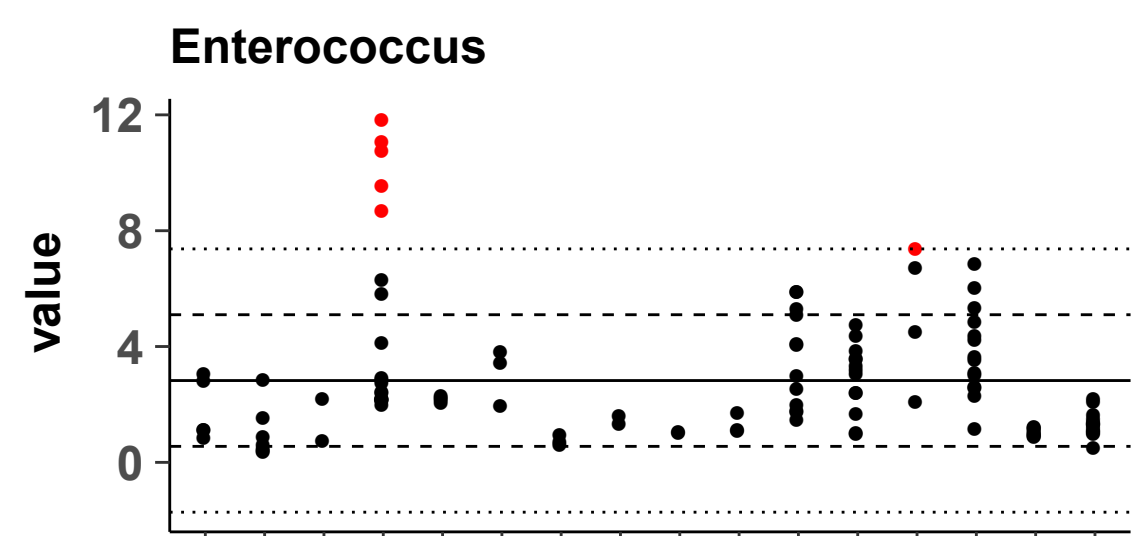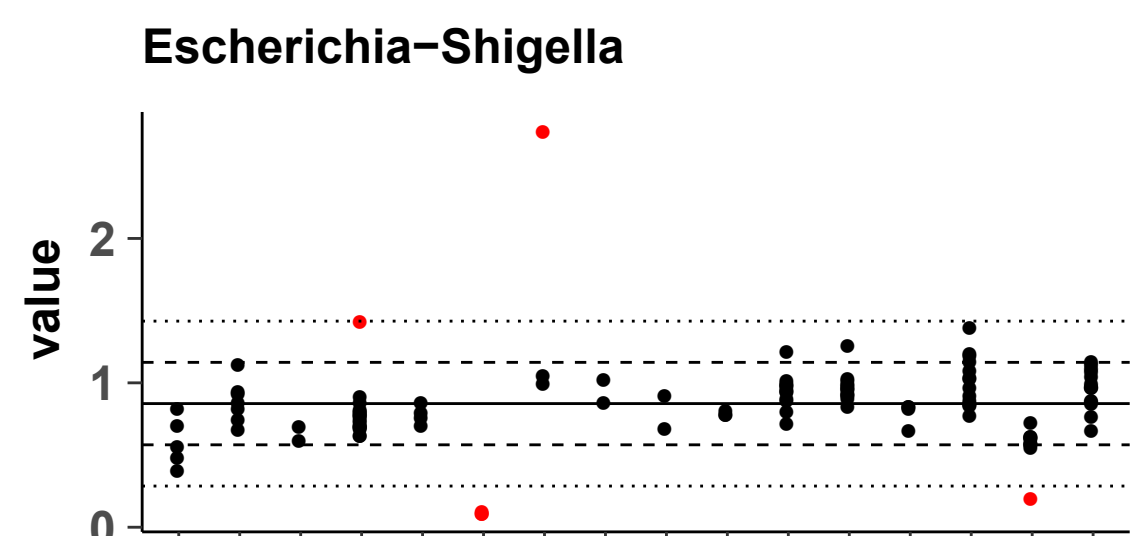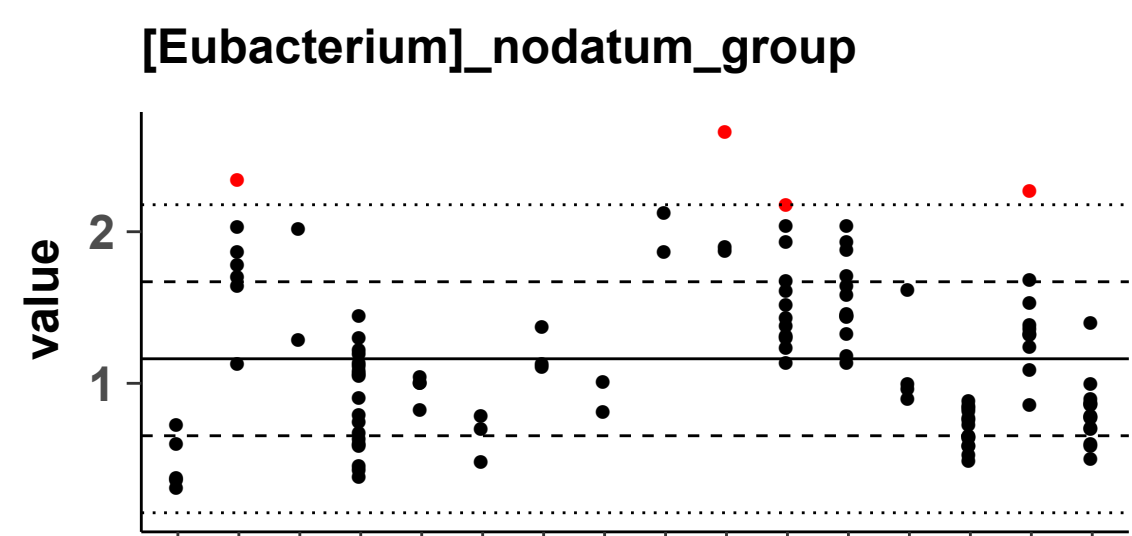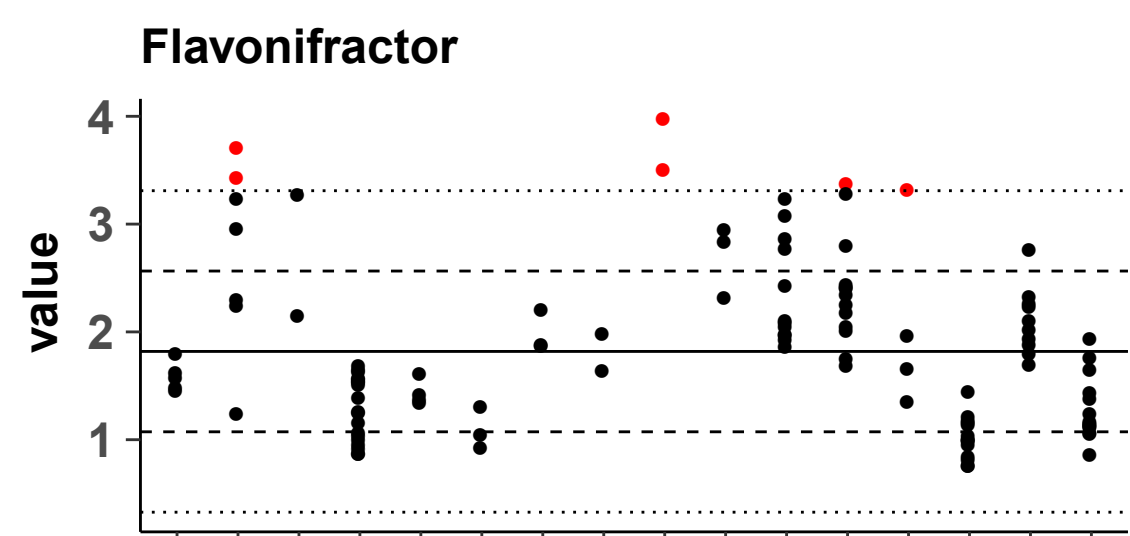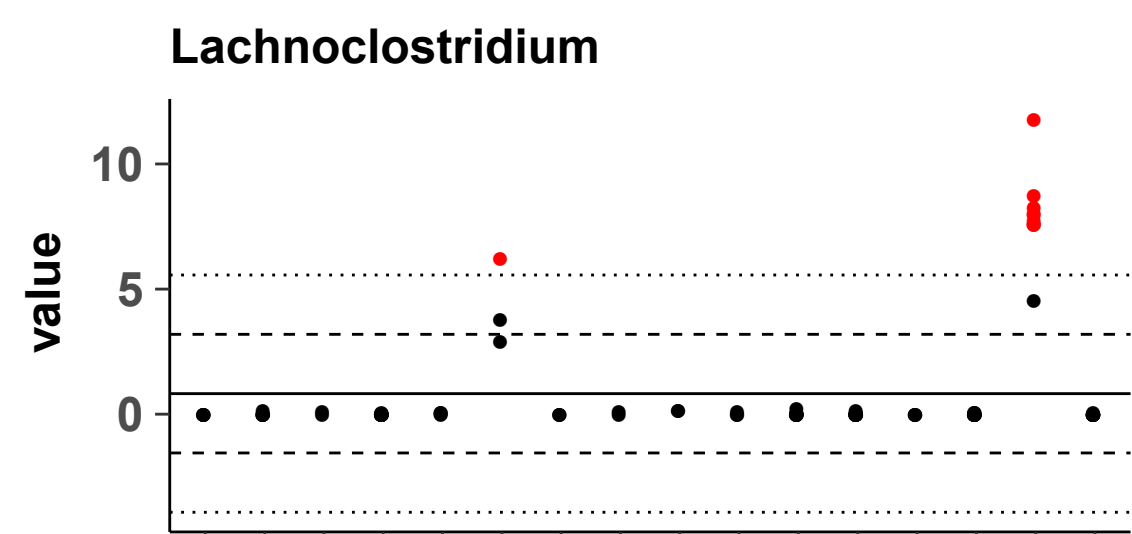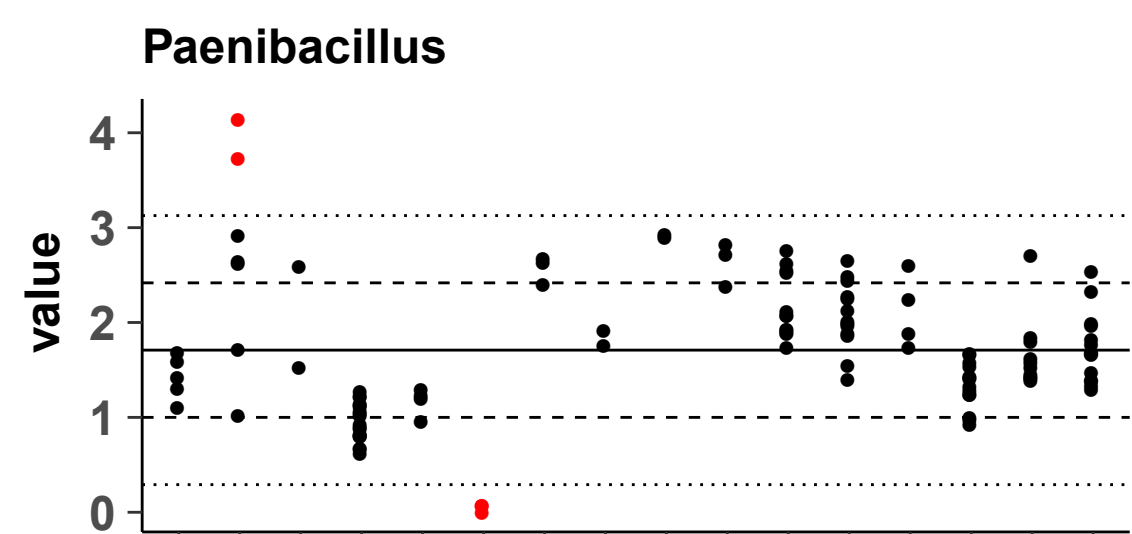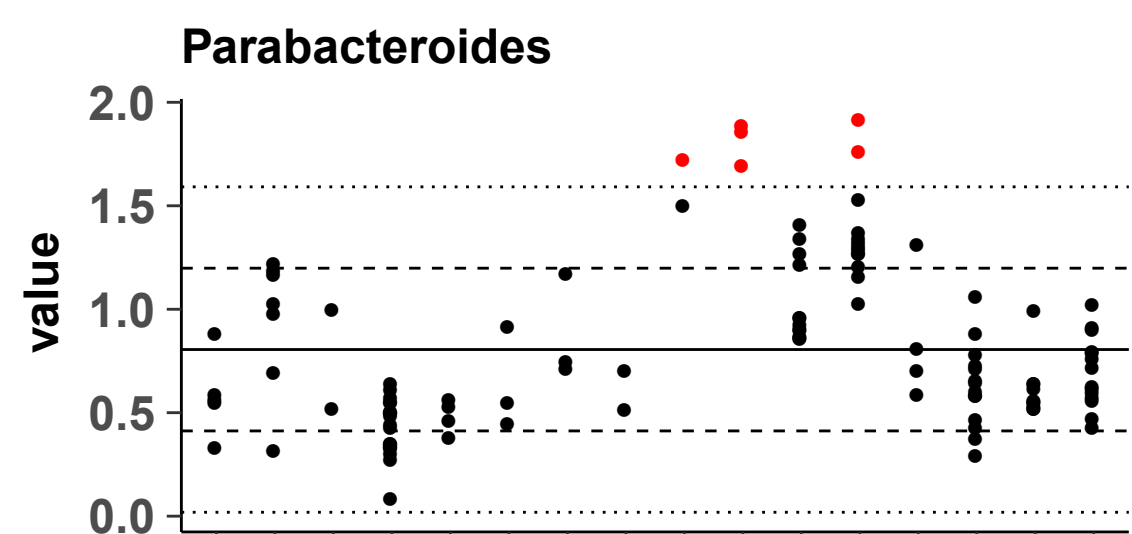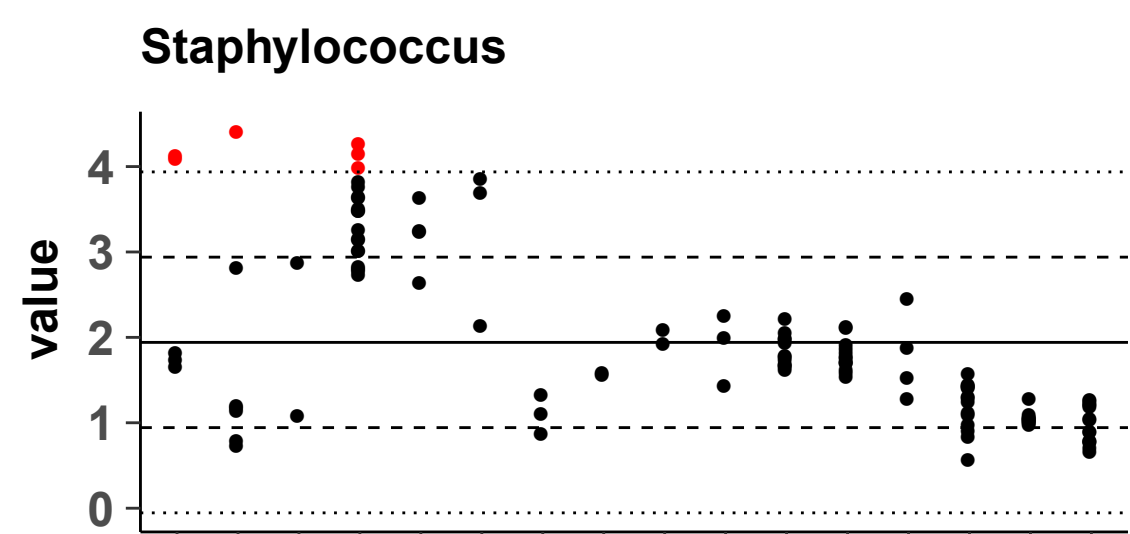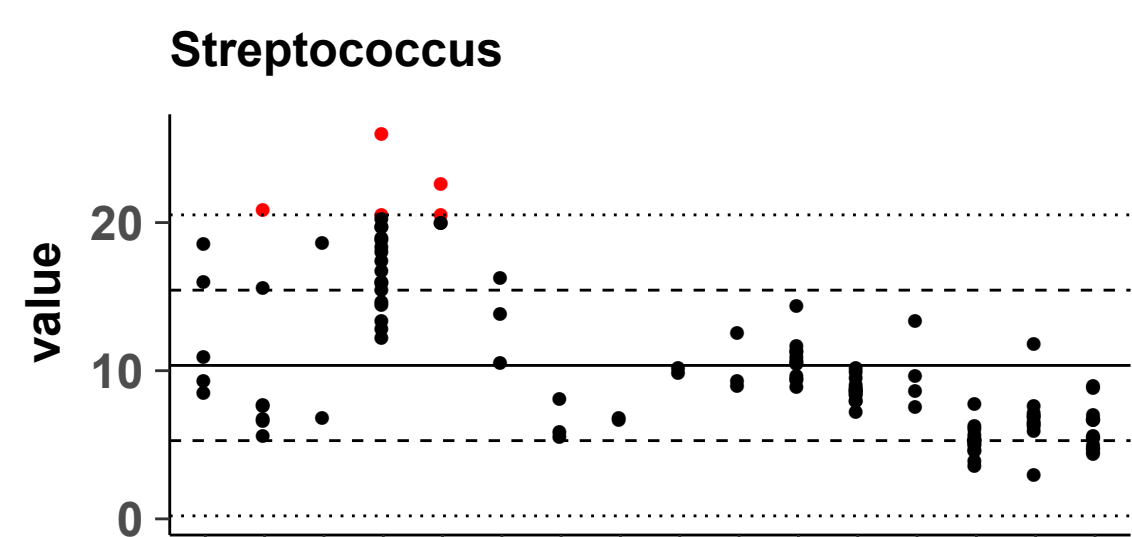

Supplement: Supplementary file 6 — Figure S4. Levey-Jennings plots for bacterial genera over the course of 16 sequencing runs (x-axis, sorted chronologically from left to right). Mean relative abundance (solid line), one standard deviation (dashed line), and two standard deviations (dotted line) are indicated. Samples in red represent observations more than two standard deviations from the mean. (PDF 972 kb) [file 40168_2018_543_MOESM6_ESM.pdf]

**a.**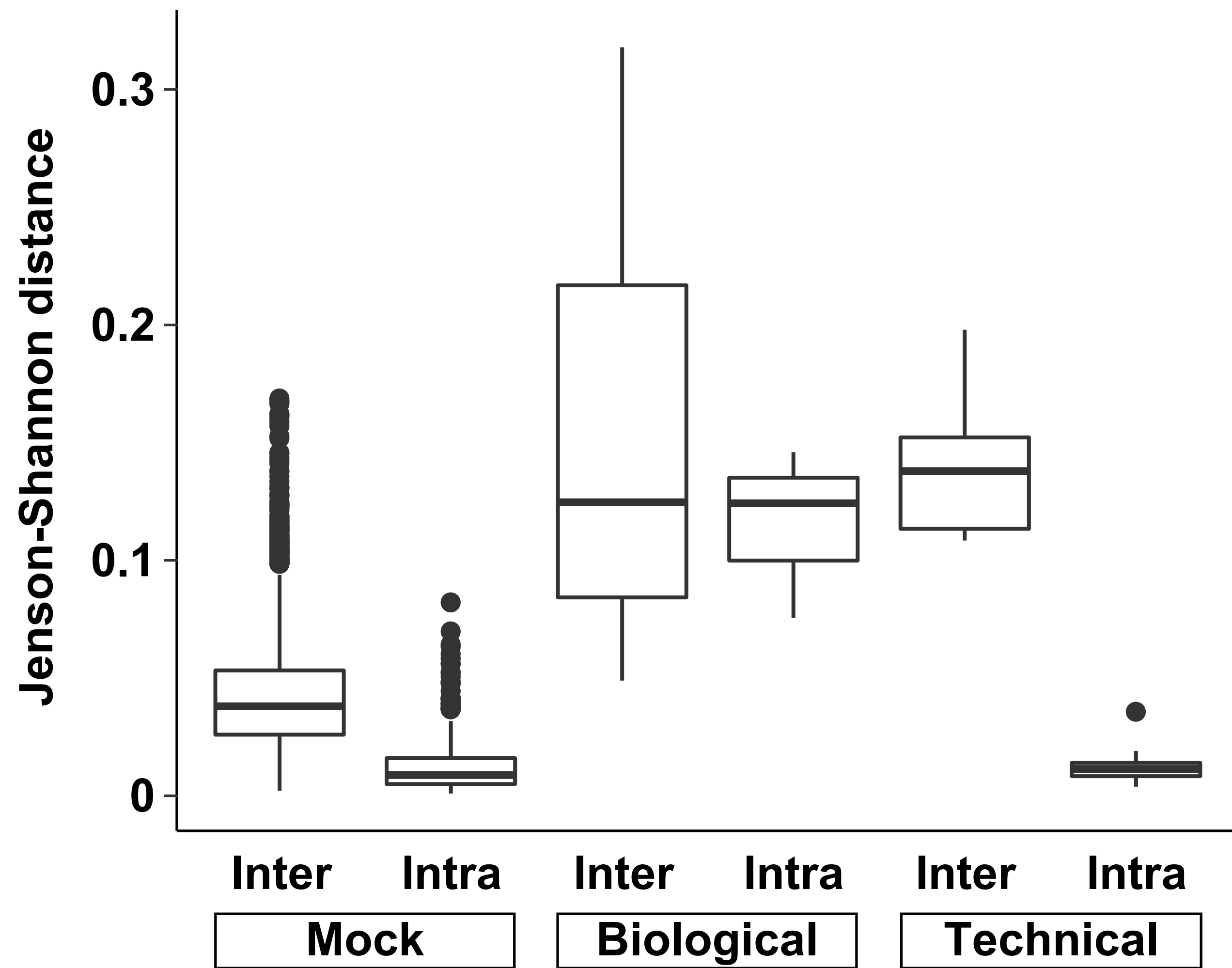**b.**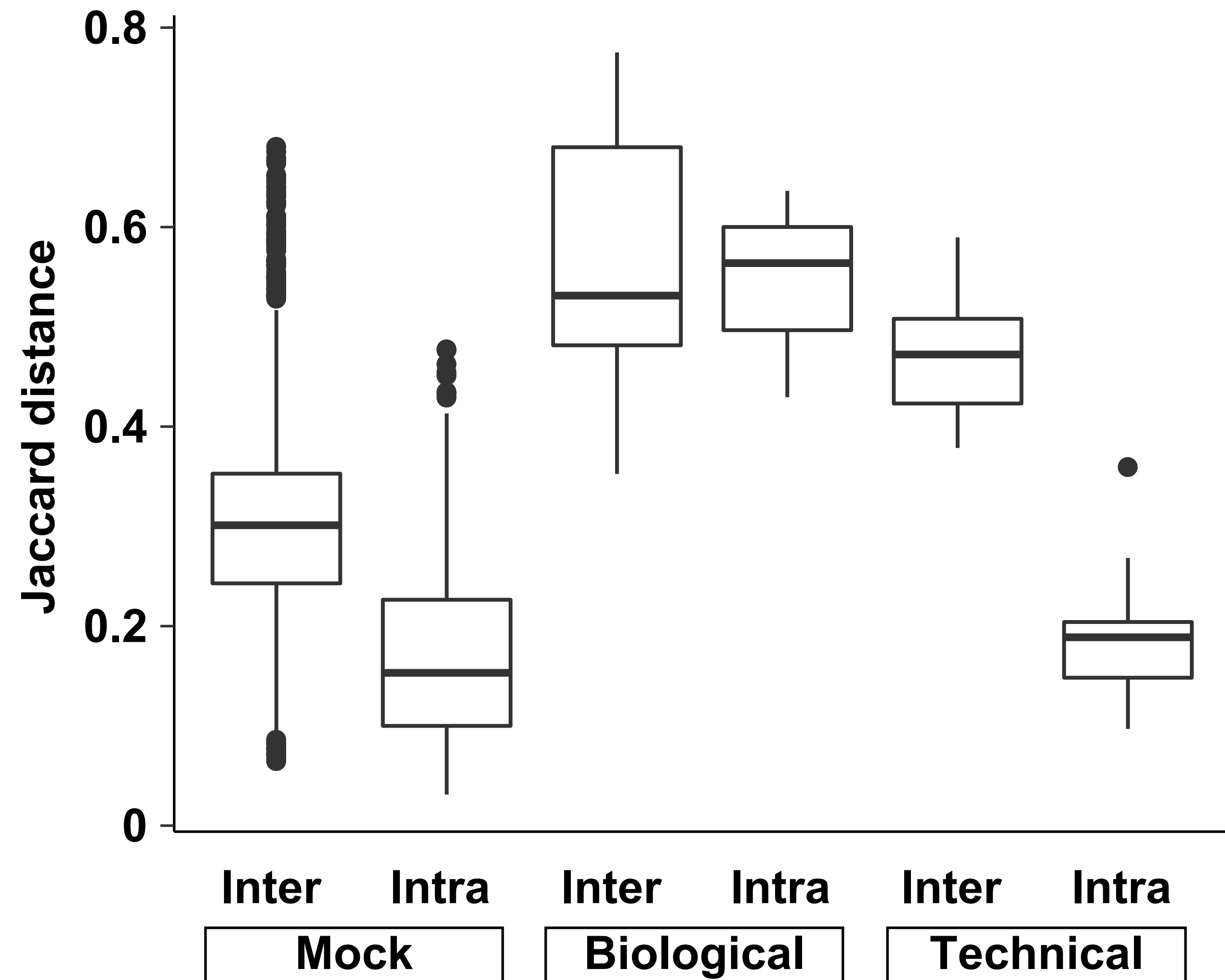

Supplement: Supplementary file 7 — Figure S5. Boxplots of Jensen-Shannon (a) and Jaccard (b) distances for bacterial mock and stool samples. (PDF 257 kb) [file 40168_2018_543_MOESM7_ESM.pdf]

a.

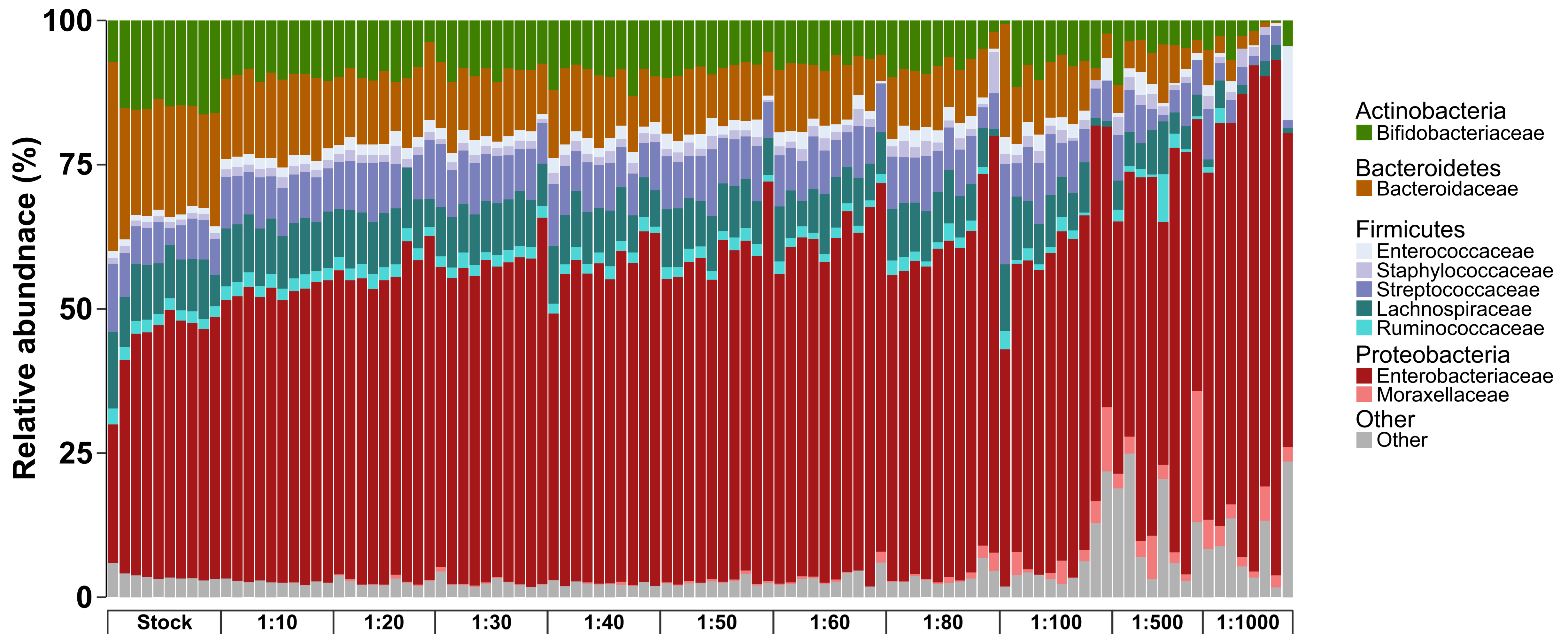

b.

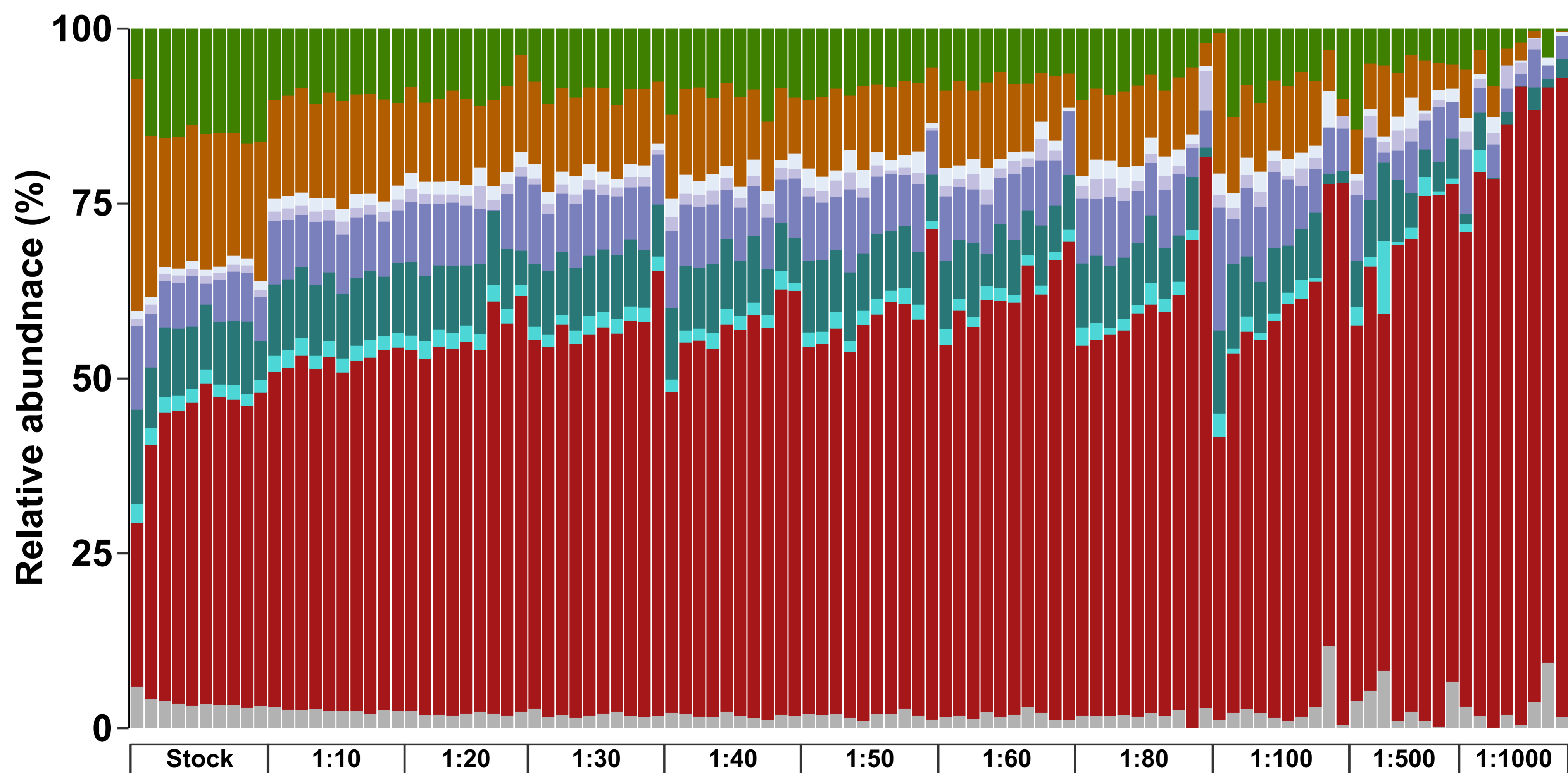

Supplement: Supplementary file 8 — Figure S6. Taxonomic composition of bacterial mock community samples pre-filtering (a) and post-filtering (b). Compositions are sorted by dilution constant and shown at the family level. Shading along bottom indicates less (darker) and more (lighter) dilution. Only taxa with an average abundance of at least 1% are shown. (PDF 253 kb) [file 40168_2018_543_MOESM8_ESM.pdf]

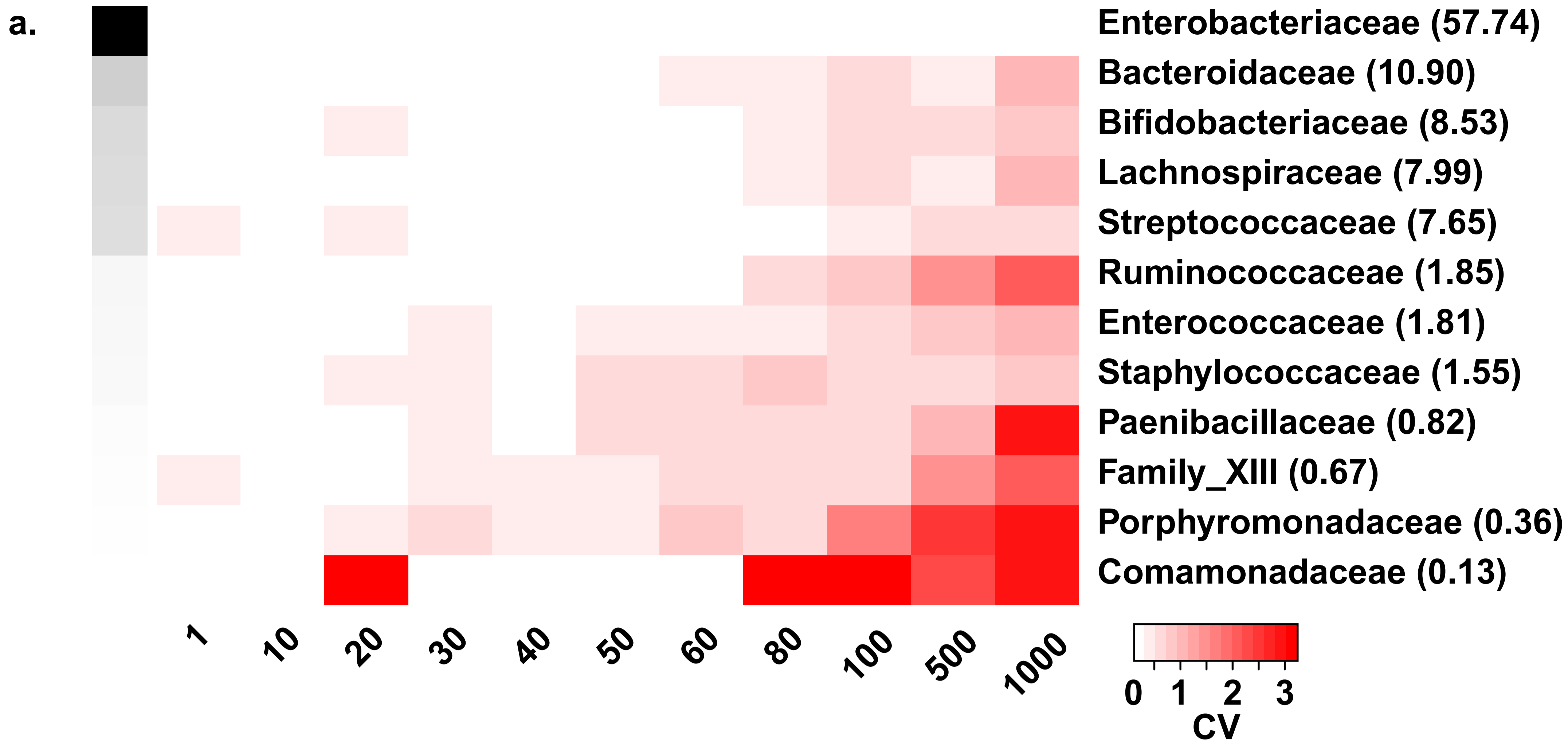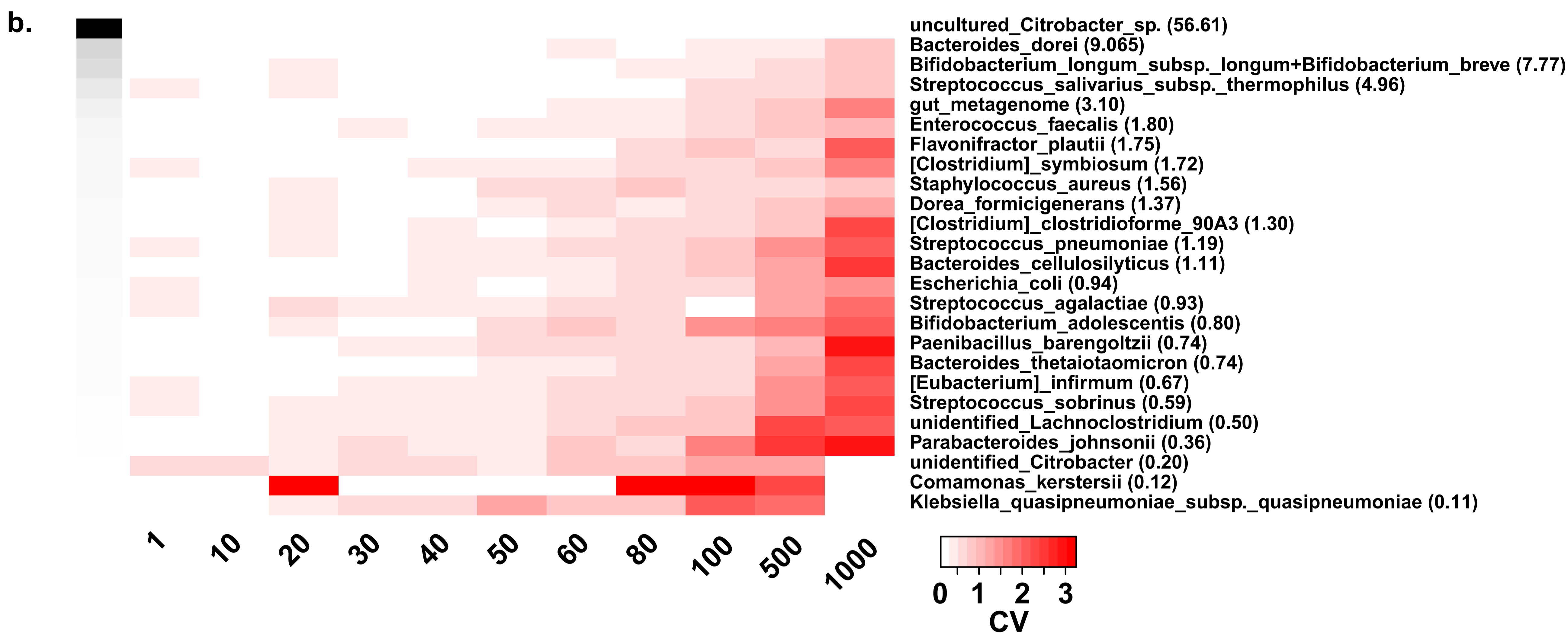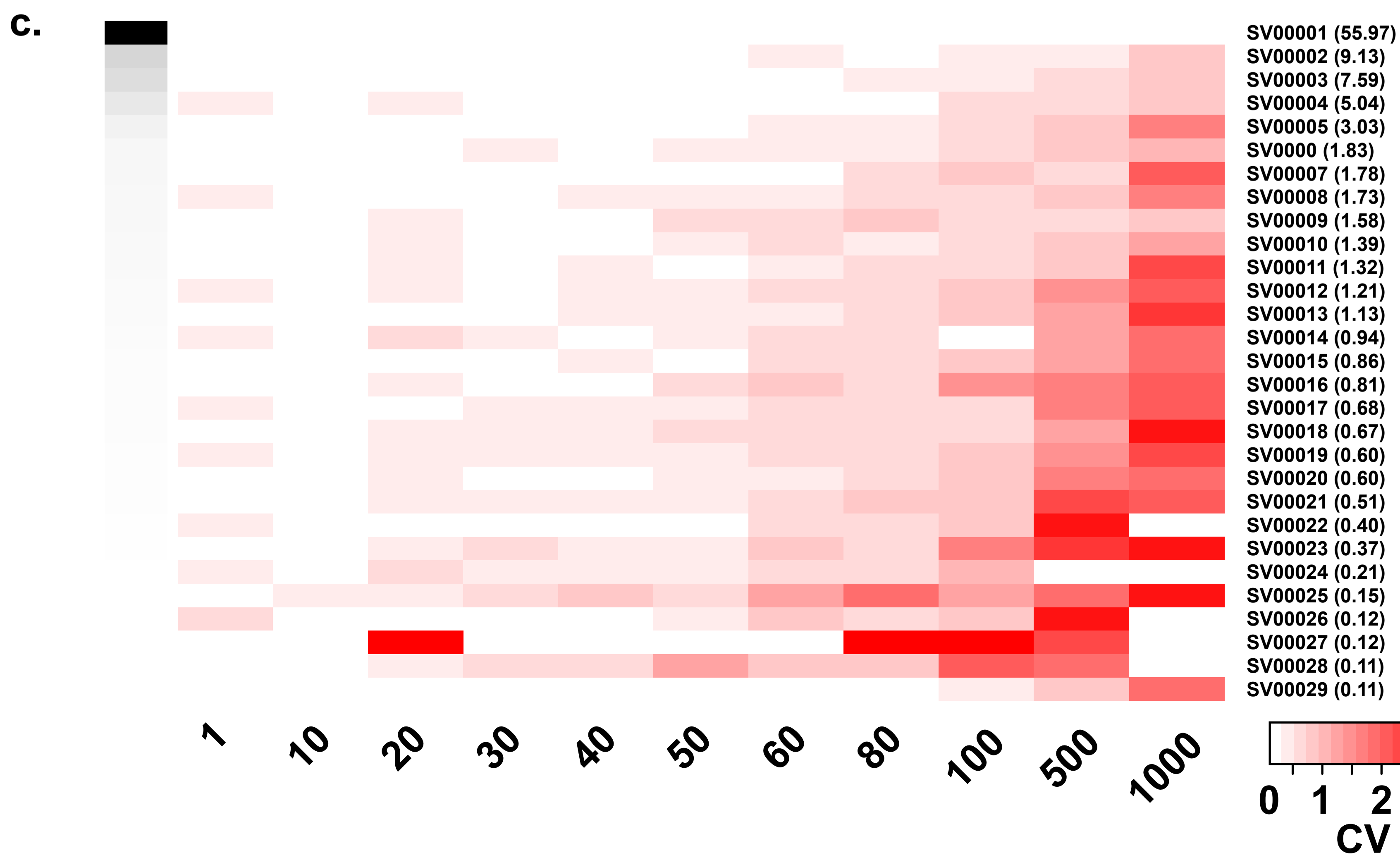

Supplement: Supplementary file 9 — Figure S7. Heatmaps of coefficient of variation (CV) values for each taxon by dilution constant, at the family (a), species (b), and sequence variant (c) levels. Greyscale cells on the left indicate mean relative abundances for each taxon (also given as percentages in parentheses). (PDF 484 kb) [file 40168_2018_543_MOESM9_ESM.pdf]
