# Supplementary material for: Quantification of variation and the impact of biomass in targeted 16S rRNA gene sequencing studies
Source: Microbiome. 2018 Sep 10;6:155. doi: 10.1186/s40168-018-0543-z (PMC6131952; doi:10.1186/s40168-018-0543-z)
Supplement: Supplementary file 3 — Table S1. Composition of the bacterial mock community. (DOCX 17 kb) [file 40168_2018_543_MOESM3_ESM.docx]

Table S1: Mock bacterial community composition and comparison to the inferred taxonomic composition from 118 samples sequenced in 16 runs.

| **Family** | **Name** | **ATCC strain** | **Comparison with Sequencing Results** | **New Column 4** | **Mis-classified** |
| --- | --- | --- | --- | --- | --- |
| Bacteroidaceae | Bacteroides cellulosilyticus | 726 | 24 SVs detected (1 *cellulosilyticus*, 11 *dorei*, 4 misclassified) | Detected | 2 *thetaiotaomicron,* 1 *coprocola*, 1 *ovatus* |
|  | Bacteroides dorei | 29 |  | 11 detected |  |
|  | Bacteroides dorei | 717 |  |  |  |
|  | Bacteroides dorei | 718 |  |  |  |
|  | Bacteroides dorei | 719 |  |  |  |
|  | Bacteroides finegoldii | 727 |  | Not Detected |  |
| Bifidobacteriaceae | Bifidobacterium adolescentis | 633 | 31 SVs detected (3 *adolescentis*, 26 *breve*/*longum*, 2 misclassified) | 3 detected | 1 *pseudolongum*, 1 *saguini* |
|  | Bifidobacterium breve | 411 |  | 26 detected |  |
|  | Bifidobacterium breve | 412 |  |  |  |
|  | Bifidobacterium longum | 845 |  |  |  |
|  | Bifidobacterium longum | 846 |  |  |  |
|  | Bifidobacterium longum | 847 |  |  |  |
|  | Bifidobacterium longum | 848 |  |  |  |
| Clostridiaceae | Clostridium clostridioforme | 306 | 18 SVs detected (2 *clostridioforme*, 4 *symbiosum,* 1 misclassified) | 2 detected | 1 *lavalense* |
|  | Clostridium clostridioforme | 317 |  |  |  |
|  | Clostridium orbiscindens | 303 |  | Not detected |  |
|  | Clostridium symbiosum | 309 |  | 4 detected |  |
|  | Clostridium symbiosum | 319 |  |  |  |
| Enterobacteriaceae | Escherichia coli | 25922 | 85 SVs detected (3 *E.* *coli*, 1 *C. freundii*) | 3 detected |  |
|  | Citrobacter freundii | 299 |  | 1 detected |  |
|  | Klebsiella pneumoniae | 700603 |  | Not detected |  |
| Enterococcaceae | Enterococcus faecalis | 29212 | 6 SVs detected (3 *faecalis*, 2 misclassified) | 3 detected | 1 *dispar*, 1 *moraviensis* |
|  | Enterococcus faecium | 700221 |  | Not detected |  |
| Family XIII Incertae Sedis | Eubacterium infirmum | 369 | 5 SVs detected (5 *infirmum*) | 5 detected |  |
| Lachnospiraceae | Dorea formicigenerans | 300 | 4 SV detected (3 *formicigenerans*) | 3 detected |  |
| Porphyromonadeaceae | Parabacteroides johnsonii | 731 | 2 SV detected (2 *johnsonii*) |  |  |
| Staphylococcaceae | Staphylococcus aureus | 25923 | 4 SVs detected (4 *aureus*) | 4 detected |  |
|  | Staphylococcus epidermis | 12228 |  | Not detected |  |
| Streptococcaceae | Streptococcus agalactiae | 12386 | 25 SVs detected (6 *agalactiae*, 2 *pneumoniae*, 3 *salivarius*, 2 misclassified) | 6 detected | 2 *sobrinus* |
|  | Streptococcus downei | 475 |  | Not detected |  |
|  | Streptococcus pneumoniae | 49619 |  | 2 detected |  |
|  | Streptococcus salivarius | 121 |  | 3 detected |  |
|  | Streptococcus vestibularis | 561 |  | Not detected |  |
